# Tuning Excitonic Properties of Monochalcogenides via Design of Janus Structures

**DOI:** 10.1021/acs.jpcc.4c01813

**Published:** 2024-07-12

**Authors:** Mateus B. P. Querne, Alexandre C. Dias, Anderson Janotti, Juarez L. F. Da Silva, Matheus P. Lima

**Affiliations:** † Department of Physics, Federal University of São Carlos, 13565-905, São Carlos, São Paulo, Brazil; ‡ University of Brasília, Institute of Physics and International Center of Physics, Brasília 70919-970, DF, Brazil; § Department of Materials Science and Engineering, University of Delaware, Newark, Delaware 19716, United States; ∥ São Carlos Institute of Chemistry, University of São Paulo, P.O. Box 780, 13560-970, São Carlos, São Paulo, Brazil

## Abstract

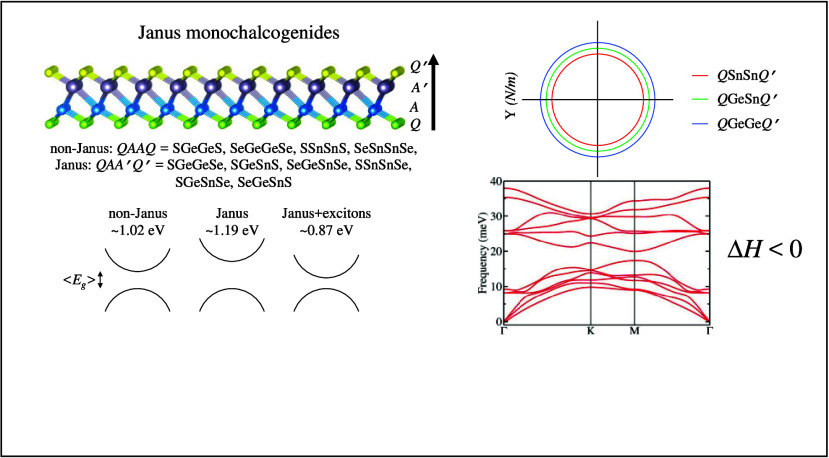

Two-dimensional (2D) Janus structures offer a unique range of properties as a result of their symmetry breaking, resulting from the distinct chemical composition on each side of the monolayers. Here, we report a theoretical investigation of 2D Janus *Q*′*A*′*AQ P*3*m*1 monochalcogenides from group IV (*A* and *A*′ = Ge and Sn; *Q*, *Q*′ = S and Se) and 2D non-Janus *QAAQ P*3̅*m*1 counterparts. Our theoretical framework is based on density functional theory calculations combined with maximally localized Wannier functions and tight-binding parametrization to evaluate the excitonic properties. The phonon band structures exhibit exclusively real (nonimaginary) branches for all materials. Particularly, SeGeSnS has greater energetic stability than its non-Janus counterparts, representing an outstanding energetic stability among the investigated materials. However, SGeSnS and SGeSnSe have higher formation energies than the already synthesized MoSSe, making them more challenging to grow than the other investigated structures. The electronic structure analysis demonstrates that materials with Janus structures exhibit band gaps wider than those of their non-Janus counterparts, with the absolute value of the band gap predominantly determined by the core rather than the surface composition. Moreover, exciton binding energies range from 0.20 to 0.37 eV, reducing band gap values in the range of 21% to 32%. Thus, excitonic effects influence the optoelectronic properties more than the point-inversion symmetry breaking inherent in the Janus structures; however, both features are necessary to enhance the interaction between the materials and sunlight. We also found anisotropic behavior of the absorption coefficient, which was attributed to the inherent structural asymmetry of the Janus materials.

## 1 Introduction

Janus structures in two-dimensional (2D) materials are distinguished by their asymmetric vertical stacking with unique surface compositions, disrupting vertical symmetry and giving rise to novel physical–chemical phenomena.^1,2^ For instance, Janus structures can be created by structural modifications within established classes of 2D materials, which includes transition-metal dichalcogenides (TMDs),^3−7^ MXenes,^8^ monochalcogenides,^9^ or any other 2D materials comprised of a few layer thickness. The symmetry breaking in the vertical direction is a key point that gives rise to numerous applications in the scope of semiconductor optoelectronic devices with prospects for applications as sensors,^10^ photocatalytic water splitting materials,^11−14^ functional coatings,^15^ and so on.

The experimental realization of Janus MoSSe^4^ has paved the way for further studies on these materials. The synthesis of Janus WSSe, MoSSe, and its heterostructures was reported using a room temperature technique, achieving high structural and optical quality by Trivedi et al.^16^ The metallic Janus MoSH monolayer was synthesized using a treatment involving H_2_-plasma by Wan et al.^17^ Through a nanoconversion technique, Hajra et al. managed to synthesize Janus BiTeX on sapphire substrates, where X = Cl, Br.^18^ Janus WSSe and its alloys were obtained by applying pulsed laser deposition by Harris et al.^19^ Practical implementations of these experiments have stimulated theoretical investigations of unproduced 2D materials for experimental verification, which can open the door to optoelectronic applications.

In this context, predicting the properties of alternative 2D Janus materials based on theoretical simulations, such as those based on density functional theory (DFT) calculations, broadens the applicability prospects of this intriguing class of materials. The enhanced transferability and prediction capability of first-principles calculations allow for the exploration of 2D Janus materials with the aim of unveiling the most promising applications for each compound. For example, Bui et al.^20^ studied Janus Ga_2_SSe, Ga_2_STe and Ga_2_SeTe, which exhibit semiconductor behavior with large dielectric constants and are therefore proposed to be efficient for use as photovoltaic absorbers and ultraviolet detectors. Janus group III monochalcogenides *A*2*QQ*′ (*A* = Ga, and In; *Q*/*Q*′ = S, Se, and Te) were investigated using first-principles calculations. These materials were shown to be suitable photocatalysts for overall water splitting by Huang et al.^21^ due to having a band gap in the range of 1.54–2.98 eV, as well as valence and conduction band edges with suitable energy values for water splitting purposes. Studies of the electronic, mechanical, and piezoelectric properties of Janus Zn*AQ*_2_ (*A* = Ge, Sn, and Si; *Q* = S, Se, and Te) were carried out with first-principles simulations by Zhang et al.,^22^ indicating promising potentials for flexible nanodevices and electromechanical systems.

The quantum confinement inherent to 2D materials enhances the electron–hole Coulomb interaction due to their reduced dielectric screening, resulting in stronger excitonic effects in comparison with 3D materials capable of being observed in experiments.^23−25^ Thus, excitonic effects must be taken into account to predict realistic properties of 2D materials.^26^ Particularly, Ridolfi et al. show how excitonic physics governs the optical response of TMD semiconductors.^27^ These electron–hole effects are particularly relevant for emerging applications in technological fields, such as valleytronics^28^ and photovoltaics.^29^

Although 2D monochalcogenide materials are highly regarded for future technologies because of their unique characteristics, the impact of breaking point-inversion symmetry in these materials by creating Janus structures is still not well understood and demands better atomic-level insights. DFT calculations are particularly suitable for this task as they can uncover the relationships among structural, electronic, and optical properties at the atomic level, particularly when considering excitonic effects.

In this work, the optoelectronic properties of 2D Janus monochalcogenides, such as SGeGeSe, SSnSnSe, SGeSnS, SeGeSnSe, SGeSnSe, and SeGeSnS in the crystal structure *P*3*m*1, are investigated using DFT calculations. Furthermore, to evaluate the excitonic properties, the Bethe–Salpeter equation (BSE) is solved using a tight-binding Hamiltonian that is parametrized based on DFT band structures and Wannier function techniques. By comparing these properties with those exhibited by non-Janus monochalcogenides (SGeGeS, SeGeGeSe, SSnSnS, and SeSnSnSe) within the crystal structure *P*3̅*m*1, our objective is to elucidate the influence of point-inversion symmetry disruption on the optoelectronic characteristics.

We verified the dynamical, mechanical, and energetic stability of all compounds, finding that the full-Janus compound SeGeSnS stands out for being the only compound with a negative value of formation energy (−0.9 meV/u.c.), indicating lower energy than the non-Janus compounds. Moreover, among all investigated Janus materials, SGeSnS and SGeSnSe have formation energies 9.1 and 32.1 meV/u.c. higher than the already synthesized Janus MoSSe, respectively, suggesting they are challenging to synthesize compared to the others. The point-inversion symmetry breaking that occurs with different *A* and *A*′ and/or *Q* and *Q*′ was quantified through Bader charge analysis due to their charge difference, where the charge transfers were well correlated with the electronegativity differences of the involved species. The electronic band structures present high electron–hole asymmetry with indirect fundamental band gaps for all compounds, where the band gaps for the Janus materials have a higher dependence on the core region than on the external sides.

Furthermore, despite the fundamental band gaps for Janus materials increasing by more than 15% compared to non-Janus materials due to point-inversion symmetry break, exciton binding energies in the range between 0.2 and 0.4 eV result in absorption coefficients that strengthen the material/sunlight interaction for Janus structures compared to non-Janus ones. This feature was demonstrated through power conversion efficiency evaluations considering both the Shockley–Queisser limit and the spectroscopy-limited maximum efficiency (SLME).^30^ The enhanced performance of Janus structures over non-Janus systems can be attributed to a synergistic interplay of point-inversion symmetry disruption and excitonic phenomena.

## 2 Theoretical Approach and Computational Details

### 2.1 Density Functional Theory Calculations

Our calculations are based on the DFT^31,32^ framework, using the all-electron projected augmented wave (PAW)^33,34^ method to describe the core–valence electron interactions, as implemented in the Vienna *Ab initio* Simulation Package (VASP),^35,36^ version 5.4.4. We used the semilocal generalized gradient approximation (GGA) proposed by Perdew, Burke, and Ernzenhof (PBE)^37^ for the exchange-correlation energy functional. Plain DFT-PBE was used for the following tasks: geometry optimizations, stability analysis through the phonon spectrum, electron density analysis through the Bader charges scheme, and density of states (DOS). However, DFT-PBE faces challenges in providing an accurate description of fundamental electronic band gaps due to self-interaction errors.^38,39^ To minimize this problem, we used the hybrid functional introduced by Heyd, Scuseria, and Ernzerhof (HSE06),^40,41^ as implemented in the VASP,^42,43^ which yields a better description of the magnitude of the fundamental band gaps compared with experimental results^44,45^ and is broadly applied for 2D systems.^46,47^

The equilibrium 2D structures were obtained through the optimization of the planar (*xy*) stress-tensor and atomic forces (*xyz*), sampling the Brillouin zone with a 9 × 9 × 1 **k**-mesh according to the Monkhorst–Pack scheme and using a plane wave cutoff energy of 520 eV.^48,49^ This cutoff energy is twice the maximum value recommended for the selected PAW projectors (Ge: 174 eV, Sn: 260 eV, S: 259 eV, and Se: 212 eV), which is required due to the slow convergence of the stress-tensor values as a function of the number of plane-waves.

Equilibrium configurations were achieved when the forces in each atom were less than 0.01 Å^–1^. A vacuum thickness of at least 15 Å separates the periodic images of the layers, avoiding interactions between them. However, for electronic and optical properties, we used a lower plane wave cutoff energy of 292 eV, which is approximately 12.5% higher than the maximum cutoff energy recommended by the selected PAW projectors and a denser **k**-mesh of 18 × 18 × 1. The high-symmetry points employed are Γ (0, 0, 0), *K* (2/3, 1/3, 0), and *M* (1/2, 0, 0), with coordinates given in units scaled by the reciprocal lattice vectors. We set the total energy criterion for the self-consistency of the electron density at 10^–6^ eV for all calculations.

### 2.2 Tight-Binding Framework

An orthogonal tight-binding (TB) Hamiltonian obtained from parametrization of maximally localized Wannier functions (MLWF), using the Wannier90 package,^50^ within the DFT-HSE06 framework feeds the WanTiBEXOS code,^51^ allowing us to calculate the optical properties by adding excitonic effects through the solution of the BSE equation. Additionally, the optical properties were derived using the independent particle approximation (IPA) to gain a clearer insight into the influence of excitons on the linear optical response. It is important to mention that analogous methods can be performed using different computational implementations.^52,53^

The Hamiltonian used in the MLWF-TB model includes *s*- and *p*-orbitals for the Ge, S, and Se atoms, while for Sn, the *s*-, *p*-, and *d*-orbitals were considered. The choice of these states was determined on the basis of DOS analysis using the DFT-PBE framework. The BSE employs a 2D truncated Coulomb potential^54^ with a 37 × 37 × 1 **k**-mesh. We selected the number of conduction and valence bands to encompass all allowed transitions within the solar emission spectrum window, namely, 0.5 up to 4.0 eV. Furthermore, we determined the power conversion efficiency (PCE) using the AM1.5G spectrum to represent solar radiance,^55^ accounting for light absorption and scattering in the atmosphere, and our methodology also incorporates solar devices operating at 298.15 K. For PCE evaluations, the layer thickness parameter includes the van der Waals distance (that is, 3.21 Å) added to the thickness calculated from the outermost atoms of the monolayers. Additional details on the BSE calculations are provided in the Supporting Information.

### 2.3 Data Analyses

In order to enhance our atomic understanding of the selection process for the Janus structure and its impact on excitonic properties, we conducted various analyses, which are described as follows.

#### 2.3.1 Phonon Bandstructure

To calculate the phonon band structures, we used the Phonopy code^56^ within the following parameters: finite-displacement of 0.010 Å, 3 × 3 × 1 supercell, the same **k**-point density, as used for the electronic properties, and a plane wave cutoff energy of 520 eV.

#### 2.3.2 Elastic Properties

We used VASP to calculate the Hessian matrix within the finite difference approach (0.015 Å), while the MechElastic code^57,58^ was used to calculate the two-dimensional elastic constant, while ElATools^59^ was used to calculate the Young’s polar modulus and the polar Poison ratio to access anysotropic elastic properties.

#### 2.3.3 Energetic Analyses

Two energetic parameters evaluate the energetic stability, namely, the formation enthalpy (Δ*H*) and the formation energy (*E*_F_). Δ*H* is calculated using the following equation:
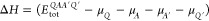
1where *Q* and *Q*′ = S or Se represent the outermost chalcogen species, whereas *A* and *A*′ = Ge or Sn represent the internal group-IV atoms; *E*_tot_^*QAA*′*Q*′^ is the total energy per unit cell of the monolayer, μ_*Q*_, μ_*A*_, μ_*A*′_, and μ_*Q*′_ represent the chemical potential for the lowest energy bulk structures, i.e., the total energy per atom for diamond-like structures for *A* and *A*′, and trigonal structures with space groups *R*3̅ and *P*3_1_21 for S and Se, respectively. The formation energy, commonly used to evaluate the energetic stability of Janus materials versus non-Janus materials,^60^ is calculated using the following equation:
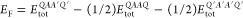
2

#### 2.3.4 Excitonic and Optical Properties

The excitonic effects in the absorption coefficient were obtained within the MLWF-TB+BSE framework,^29,61^ as implemented in the WanTiBEXOS code.^51^ The structure of the exciton band allows for the identification of whether the ground state of the exciton is direct or indirect. The absorption coefficients were obtained from both the BSE and the IPA levels to demonstrate the role of excitonic effects in the description of the absorption spectrum.

#### 2.3.5 Power Conversion Efficiency

We estimated the PCE using both the SLME method^30^ and the Shockley–Queisser upper limit (SQ-Limit),^62^ that was fitted from the band gap value. A postprocessing step evaluated the PCE from the output of the WanTiBEXOS code. Additional details of the mathematical formalism of both methods are discussed elsewhere.^63^

## 3 Results and Discussion

The findings are systematically categorized into three distinct sections to improve clarity and coherence. Initially, we elucidate and deliberate on the optimized configurations (Section 3.1) to verify their authenticity and replicability. Subsequently, we characterize the stability of all compounds (Section 3.2) to confirm their feasibility. Ultimately, we disclose the optoelectronic attributes (Sections 3.3 and 3.4) to explore their distinctive properties and potential applications.

### 3.1 Optimized Structures

In this section, we provide a classification of the structures as non-Janus, internal-Janus, external-Janus, and full-Janus. We validate our calculations by comparing the calculated lattice parameters with published data. In addition, we discuss the most significant geometric parameters.

#### 3.1.1 Structures Classification

**Figure 1:**
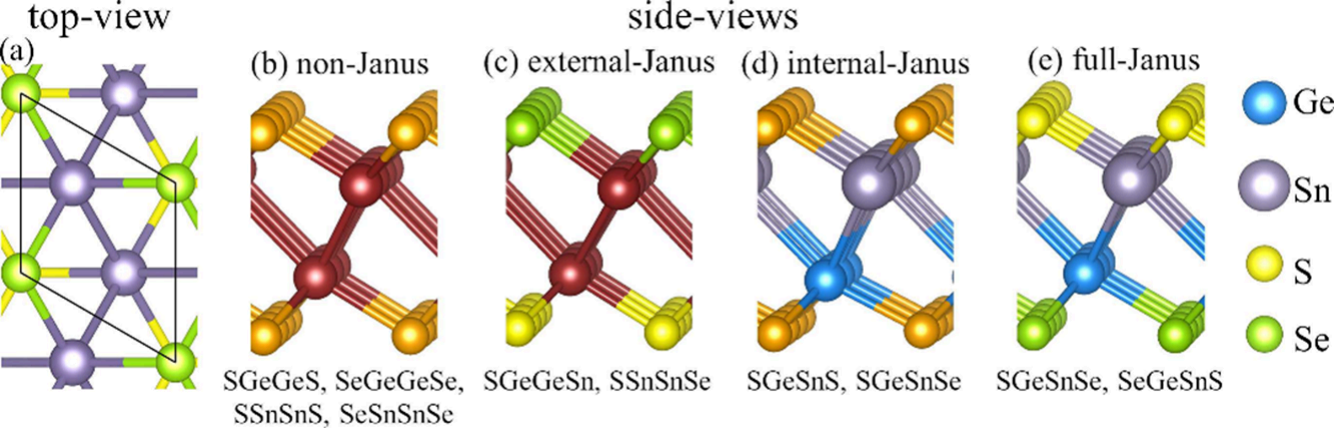
Ball-and-stick models of Janus monochalcogenides structures. The left panel shows the top view for the *P*3*m*1 structure, analogous to all other compounds investigated. The other panels show side views of the non-Janus, external-Janus, internal-Janus, and full-Janus structures. The red atoms represent the core region composed of either Ge or Sn, and the atoms in gold, the atoms in the external layers consisting of either S or Se. The compositions are written considering the atoms from bottom to top.

Figure 1 depicts ball-and-stick models for selected 2D monolayers, which have a hexagonal unit cell, as shown in Figure 1a. These structures originate from previously investigated centrosymmetric group-IV monochalcogenides with space group *P*3̅*m*1.^64−67^ However, the formation of Janus structures breaks the inversion-center symmetry (which is classified through the use of the Atomic Simulation Environment (ASE) tool^68^) resulting in novel optoelectronic characteristics.^69^ Each material comprises four atomic layers, with two triangular layers of group-IV atoms sandwiched between triangular chalcogen lattices. Thus, the point-inversion symmetry breaks should be imposed by mixing the composition of two central group-IV layers, mixing the composition of the outermost chalcogen layers, or both alternatives.

In this work, we investigate four classes of structures, namely, (*i*) non-Janus, that is, the structures that preserve the point-inversion symmetry, as shown in Figure 1b; (*ii*) external-Janus, which breaks the point-inversion symmetry by mixing the outermost chalcogen layers, as shown in Figure 1c; (*iii*) internal-Janus, which mixes the composition of the internal layers, as indicated in Figure 1d; and (*iv*) full-Janus structures, in which each layer has a different composition (see Figure 1e). The possible chemical compositions for each one of these flavors are indicated below each panel (b–e).

#### 3.1.2 Equilibrium Lattice Parameters

First, we validate our calculations by comparing our equilibrium lattice parameters with available published data.^67,70,71^ The lattice parameters for non-Janus structures shown in Table 1 are in good agreement with the reported values of 3.65 Å for SGeGeS, 3.81 Å for SeGeGeSe, 3.95 Å for SSnSnS, and 4.09 Å for SeSnSnSe obtained from DFT simulations at the same level as those adopted in our work.

**Table 1:** Structural Data^a^

system	*a* (Å)	*d*_*QA*_ (Å)	*d*_*AA*′_ (Å)	*d*_*A*′*Q*′_ (Å)	θ_*AA*′_ (°)	*c*_11_ (N m^–1^)	*c*_12_ (N m^–1^)	Δ*H* (eV/u.c.)	*E*_F_ (meV/u.c.)
SGeGeS	3.65	2.45	2.97	2.45	75.9	80.82	26.31	–1.20	0
SeGeGeSe	3.81	2.58	2.99	2.58	79.1	75.94	20.55	–1.44	0
SSnSnS	3.95	2.63	3.38	2.63	71.4	50.81	18.80	–1.64	0
SeSnSnSe	4.09	2.76	3.37	2.76	78.8	48.48	14.72	–1.57	0
SGeGeSe	3.73	2.47^b^	2.98	2.56^c^	77.6	77.85	23.86	–1.10	22.5
SSnSnSe	4.02	2.65^d^	3.37	2.75^e^	73.1	51.15	63.06	–1.59	11.3
SGeSnS	3.81	2.48^b^	3.17	2.61^d^	73.9	63.82	24.68	–1.38	45.3
SeGeSnSe	3.95	2.61^c^	3.18	2.74^e^	76.8	61.67	20.25	–1.28	29.6
SGeSnSe	3.88	2.50^b^	3.18	2.72^d^	75.1	59.32	24.63	–1.27	68.3
SeGeSnS	3.88	2.59^c^	3.17	2.62^d^	75.5	63.06	19.64	–1.34	–0.9

a Lattice parameter, *a*; Bond length between *Q*–*A* (*d*_*QA*_), *A*–*A*′ (*d*_*AA*′_), and *A*′–*Q*′ (*d*_*A*′*Q*′_) first neighbor sites; Angle between *A*–*A*′–*A* sites (θ_*AA*′_) same for *A*′–*A*–*A*′; Elastic constants *c*_11_ and *c*_12_; Energetic properties, i.e., formation enthalpy Δ*H* and formation energy *E*_F_ given by eqs 1 and 2, respectively, for *QAA*′*Q*′ structures (*A* and *A*′ = Ge and/or Sn; *Q* and *Q*^′^ = S and/or Se). The horizontal lines in the table separate classes of materials following the order non-Janus → external → internal → full-Janus.

b Ge–S.

c Ge–Se.

d Sn–S.

e Sn–Se.

To our knowledge, values for the lattice parameters of Janus *P*3*m*1 2D layers are lacking in the literature. Thus, we rationalize the lattice parameters for the Janus structures as averages of the non-Janus ones. Specifically, we contrasted the calculated equilibrium lattice parameter for Janus *QAA*′*Q*′ (*a*_0_^*QAA*′*Q*′^) with averaged lattice parameters of *QAAQ* and *Q*′*A*′*A*′*Q*′ non-Janus structures by
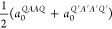
3

We used the average lattice parameter of the non-Janus as the initial lattice parameter for the Janus structures. After optimizing the structure, we found discrepancies shorter than 0.01 Å, demonstrating that the averages of the non-Janus lattice parameters can be used accurately to determine the lattice parameters for Janus monolayers. This particular relation corresponds to a null bowing parameter in the context of alloys.^72^ We attribute this behavior to a well-correlated relationship between the lattice parameter and the internal coordinates (such as the lengths and angles of the bonds between them), considering that the atoms Ge and S have shorter radii than the Sn and Se, respectively.^73^

#### 3.1.3 Equilibrium Internal Lattice Parameters

Interestingly, the bond lengths for the same atomic pair species and the angles between them slightly vary for distinct compounds, despite the null bowing parameter. For example, the pair Ge–S has a bond length of 2.45 Å in non-Janus materials and varies up to 2.50 Å in Janus structures (others shown in Table 1). However, these bond lengths are not the only important parameters. The buckling angle θ_*AA*′_ adjusts itself, resulting in a null bowing parameter. For example, SGeSnS and SeGeSnSe present similar Ge–Sn distances, varying only 0.01 Å; however, θ_*AA*′_ changes with 2.9°. Thus, both the bond lengths and the buckling angles adjust synergistically to nullify the bowing parameter.

### 3.2 Structural Stability

We investigate the stability of those materials through three complementary properties: (*i*) Phonon spectra, which provide information on dynamical stability; (*ii*) Elastic properties related to mechanical stability; (*iii*) Energetic stability characterized by both formation enthalpy and formation energy.

#### 3.2.1 Dynamical Stability via Phonons Calculations

**Figure 2:**
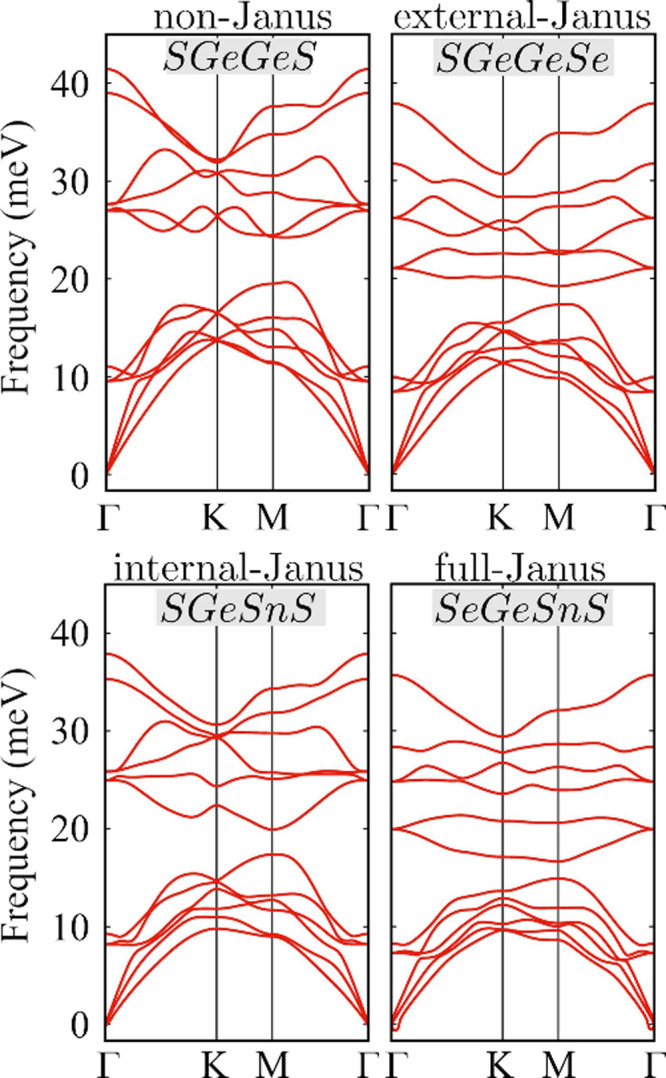
Phonon band structures for non-Janus and external-Janus on the top panels; internal-Janus and full-Janus on the bottom panels.

Representative phonon band structures for the non-Janus, external-Janus, internal-Janus, and full-Janus structures are shown in Figure 2, while the remaining phonon data are summarized in the SI. For all structures, we obtained real (nonimaginary) frequencies for all acoustic branches, indicating dynamical stability. Furthermore, the three acoustic branches have a linear shape around the Γ-point (ω ∝ |**k**|),^99^ and we did not observe low-frequency parabolic valleys (ω ∝ |**k**|^2^) previously reported for phosphorene-like Janus monolayers.^60^ Instabilities can occur under external conditions, such as strain, when low-frequency parabolic valleys are present, as stated by Gan et al.^100^ Therefore, the *P*3*m*1-derived Janus monolayers display high phonon stability due to their linear acoustic branches.

As all unit cells have four atoms, there are 12 phonon branches each. All structures present a low-frequency band composed of three optical branches and three acoustic branches coexisting in the same frequency region, opening the possibility for strong optical-acoustic scattering. These low-frequency bands are separated from the high-frequency bands composed of six optical branches, where the maximum frequencies range from 30 to 40 meV and fall in the same range as other 2D chalcogenides,^64,74,75^ but are around 5× lower than those of h-BN and graphene.^76^

#### 3.2.2 Mechanical Stability

The stress–strain (σ–ε) relation for hexagonal 2D materials is given by^77^
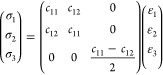
4Thus, there are only two independent elastic constants, shown in Table 1, which provide information on mechanical stability.

**Figure 3:**
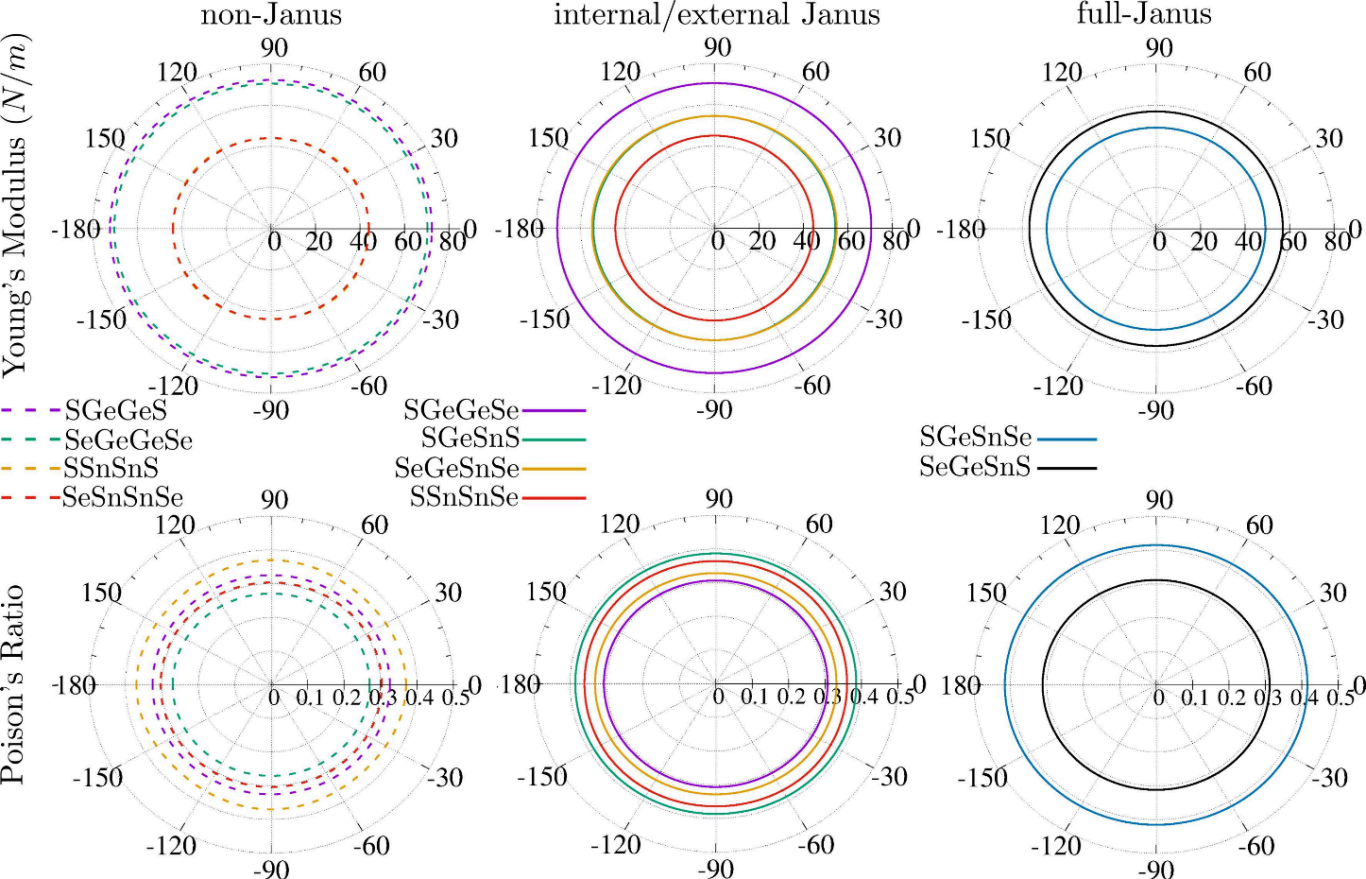
Angle-resolved Young’s modulus (on the top) and Poisson’s ratio (on the bottom) for all selected non-Janus and Janus materials.

The mechanical stability criterion presented by Maździarz^78^ is *c*_11_ – *c*_12_ > 0 and *c*_11_ + *c*_12_ > 0. These relations are satisfied by all compounds, demonstrating that these materials are mechanically stable. Furthermore, *c*_11_ decreases as the lattice parameters increase, which is consistent with a lower rigidity for materials with longer bond lengths. Figure 3 presents the angular dependence of Young’s modulus and Poisson’s ratio. The perfect circular shape confirms the isotropic elastic properties expected for hexagonal 2D materials. The left panel shows the non-Janus structures, with the compound SGeGeS being the most rigid one, with a Young’s modulus of 72.3 N m^–1^, and the compound SeGeGeSe being the second most rigid, with a Young’s modulus of 70.3 N m^–1^, while the less rigid compounds SSnSnS and SeSnSnSe have a Young’s modulus of 44.0 N m^–1^. These results indicate that the composition of the core region dictates the hardness of these materials.

The core region also dominates the elastic properties, with SGeGeSe being the most rigid compound with a Young’s modulus of 70.5 N m^–1^. The two internal Janus structures present similar values for the Young’s modulus, around 55.0 N m^–1^, while different external Janus structures, e.g., SSnSnSe, corroborates the fact that the core region defines the hardness of these materials, exhibiting a Young’s modulus of 44.4 N m^–1^, close to the reported value of 40.0 N m^–1^ for stanene.^79^ The full-Janus SeGeSnS presents the highest Young’s modulus among these structures (56.9 N m^–1^), being only 7.8 N m^–1^ higher than SGeSnSe, shows that the external bonds only weakly influence the hardness of the material.

#### 3.2.3 Energetic Stability

The energetic stability is initially examined by calculating the formation enthalpy per unit cell, as listed in Table 1. All systems exhibit a negative formation enthalpy, indicating an exothermic process. Among the different structures, the non-Janus configurations have the lowest formation enthalpy per unit cell, with an average value of −1.46 eV/u.c. However, external-Janus and internal-Janus structures have an average formation energy of −1.34 eV/u.c., while full-Janus structures have a formation energy of −1.30 eV/u.c.. The slight differences of 0.12 and 0.16 eV, respectively, between the selected non-Janus and Janus materials suggest that the Janus structures have formation energies comparable to those of the pristine ones, indicating that both are energetically competitive.

Table 1 also shows positive (endothermic) formation energies (*E*_F_) for almost all investigated materials, with only one material (SeGeSnS) showing negative (exothermic) formation energies. These values indicate that the corresponding non-Janus monolayers are more energetically favorable. However, these values fall in the same range as other Janus monolayers predicted to be energetically stable.^60^ For comparison, we also calculated a formation energy of 36.2 meV/u.c. for the MoSSe Janus monolayer that was demonstrated experimentally^4,80^ and a comparison between this result and the *P*3*m*1 Janus monolayers investigated in this work demonstrate that SGeGeSe, SSnSnSe, SeGeSnSe, and SeGeSnS have a lower formation energy than the one already synthesized experimentally. However, SGeSnS and SGeSnSe have higher formation energies by 9.1 meV/u.c. and 32.1 meV/u.c., respectively, suggesting that these structures should be challenging to grow experimentally compared to the other Janus structures.

### 3.3 Symmetry-Breaking Effects on the Electronic Structure

Upon validation of the stability of the non-Janus and Janus monolayers through a comprehensive assessment of their vibrational, elastic, and energetic properties, we advance to an in-depth analysis of their electronic properties. This entails a comparative analysis of Janus materials against their non-Janus analogues to quantify the influence of Janus structures on the properties of 2D materials. To accomplish that, we used the DFT-HSE06 framework for a precise evaluation of electronic band structures, alongside the BSE approach to account for excitonic phenomena.

#### 3.3.1 Composition of Valence and Conduction States

**Figure 4:**
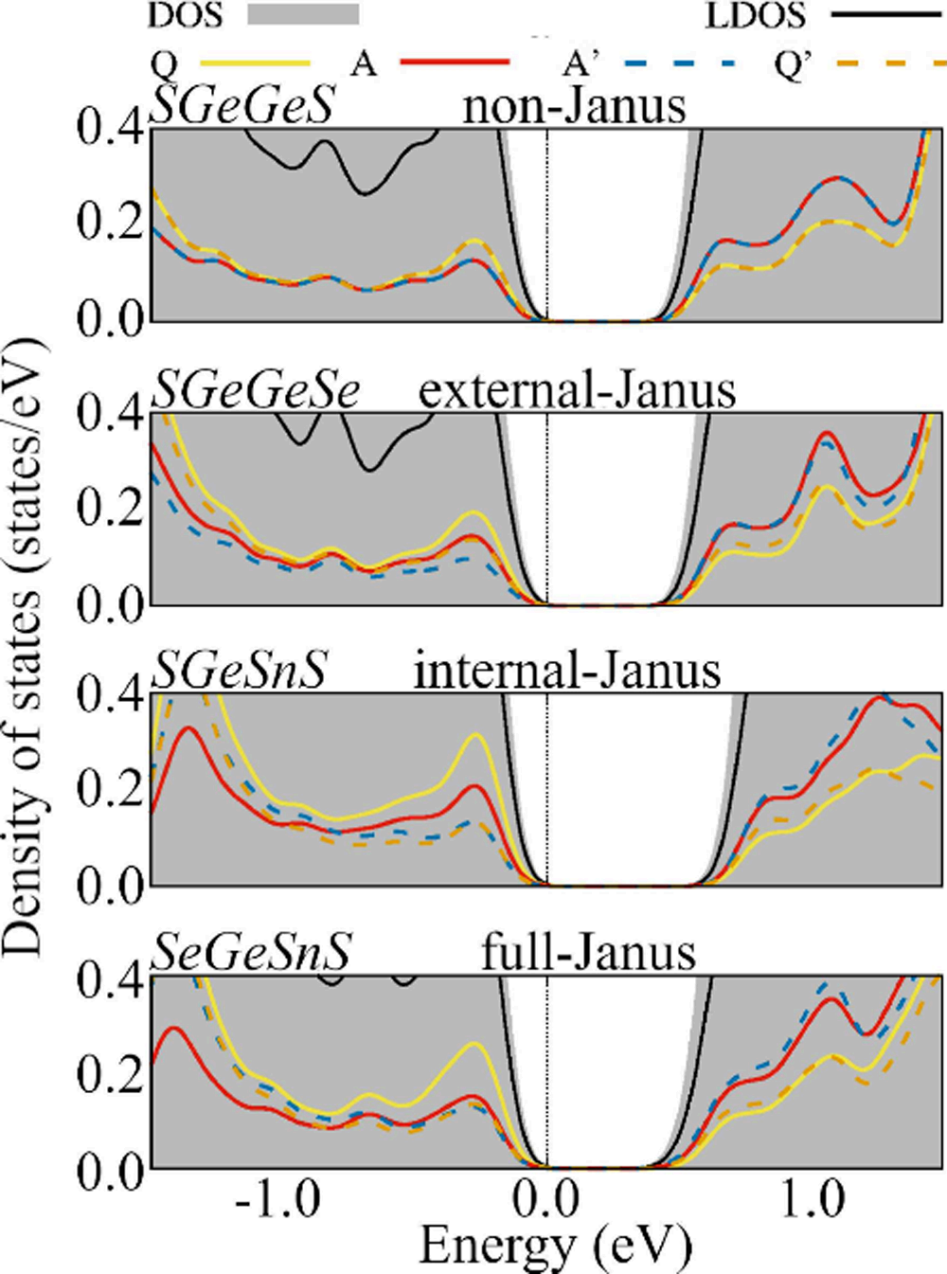
Density of states (DOS) evaluated within the DFT-PBE framework. Each panel presents the total DOS, the sum of the local DOS in all atoms (LDOS); and local DOS at *Q*, *A*, *A*′, and *Q*′. The VBM is set to zero. The DOS for all the other materials investigated in the present work are contained in the SI.

Figure 4 presents the total and local DOS per unit cell for the representative systems. All systems have Bloch functions hybridized over all atomic species with similar contributions at both the valence-band maximum (VBM) and the conduction-band minimum (CBM). However, VBM has a slightly higher contribution of the chalcogen atoms, whereas the contribution of group-IV atoms is slightly enhanced at CBM. This result is consistent with the higher electronegativity of chalcogen atoms compared to group-IV ones, inducing an electron flow from Ge and Sn to S and Se.

Janus materials lose their point-inversion symmetry, leading to signatures in the LDOS. It is possible to note in Figure 4 a higher contribution of sulfur atoms than selenium for VBM is observed in the external monolayers of Janus (SGeGeSe and SSnSnSe); however, an inverse trend is observed close to CBM. Internal-Janus monolayers (SGeSnS and SeGeSnSe) have a higher contribution from Sn than from Ge close to the CBM, and the charge transfer in the pair SnS is greater than in the pair GeS as a consequence of the lower electronegativity of Sn compared to Ge. Full-Janus monolayers (SeGeSnS and SGeSnSe) sum up all these effects, presenting different contributions for each species.

#### 3.3.2 Atomic Charge Transfer

**Figure 5:**
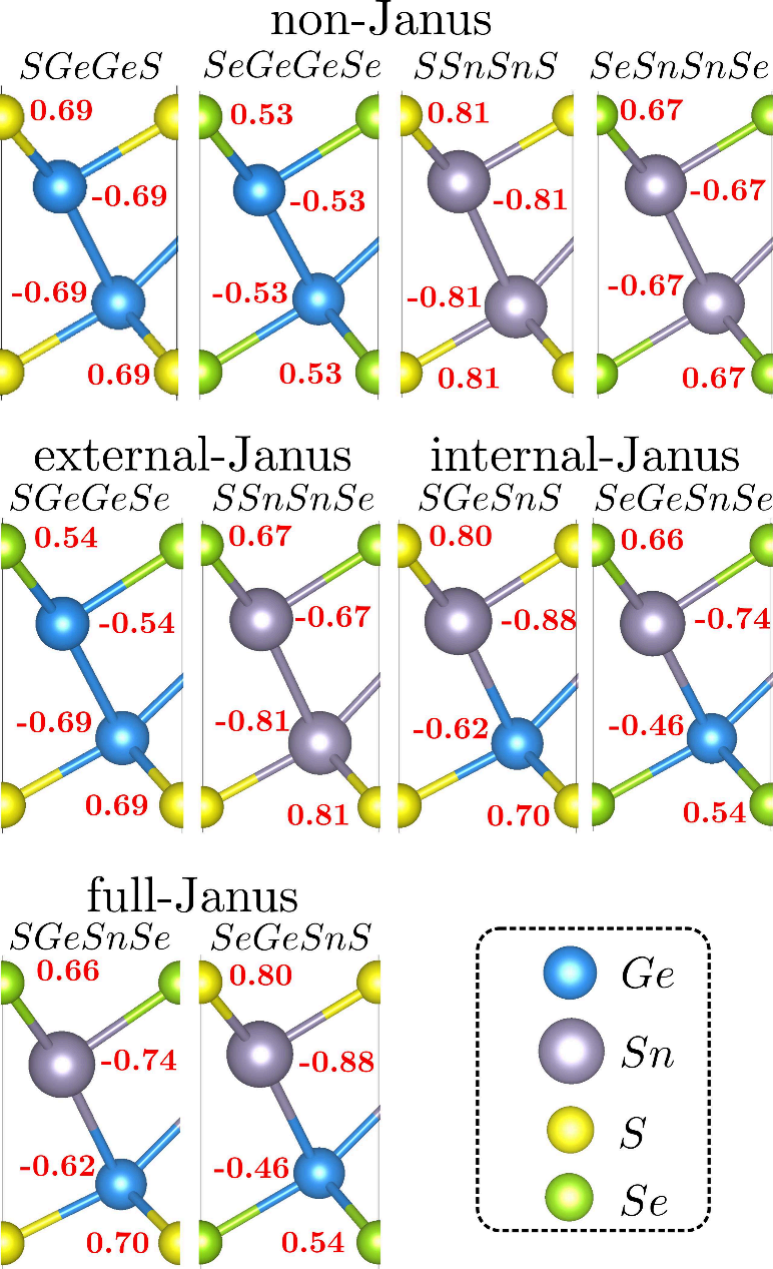
Charge excess calculated from a Bader charge analysis in units of the elementary charge *e* are shown in red. Positive (negative) values represent an excess of electrons (holes).

Because local DOS distinctly reveals the implications of electronegativity disparities, we meticulously investigated the charge dynamics between the atomic species. Bader charge analysis elucidates the charge transfer phenomena between atomic species, as depicted in Figure 5, underscoring the consequences of disrupting point-inversion symmetry. Electronegativity differences mostly dominate charge transfer between chalcogens (*Q*) and group-IV (*A*) atoms. Considering all Janus and non-Janus materials, we find charge transfers where *A* loses charge in the range of 0.46*e* to 0.88*e*, which agrees with other 2D materials already studied (e.g., each sulfur receives 0.62*e* in WS2^81^), indicating the presence of Coulomb interactions in the *A*–*Q* chemical bonds. These values are consistent with other group-IV Janus chalcogenides.^82^ One can observe symmetric charge transfer from the inner group-IV atoms (*A*) to the chalcogen atoms (*Q*) for the non-Janus compounds, resulting in the absence of a built-in electric field. These compounds exhibit charge transfers between *AQ* dimers with values ranging from 0.53*e* to 0.81*e*.

However, the symmetry-breaking effects presented by the 2D Janus *QAA*′*Q*′ materials occur due to different charge flows between *AQ* and *A*′*Q*′ groups, ranging from 0.54*e* to 0.80*e* in the following order: GeSe → SnSe → GeS → SnS. It is worth noting that particular features of the *P*3*m*1 structure impose a charge flow in the *AQ* groups in a direction opposite to those in the *A*′*Q*′ groups. Thus, the composition plays a key role in the built-in electric fields as it determines the charge transfer strengths.

#### 3.3.3 Built-in Electric Fields

Built-in electric fields are effective electric fields that are often utilized to elucidate the origin of polarization resulting from the breaking of point-inversion symmetry in Janus materials.^83^ This electric field is defined as the difference between the internal electric field of pristine (non-Janus) materials and those of Janus materials.

These charge transfers give rise to built-in electric fields. As the charge transfer between Ge and Sn is lower than the charge transfer between group IV and chalcogenides, the latter dominates the built-in electric fields. The *AQ* (*A*′*Q*′) groups generate electric fields between *A* and *A*′ sites pointing from *A* (*A*′) toward the central region of the monolayer. The resultant built-in electric field arises from the interplay between the electric fields generated by these *AQ* and *A*′*Q*′ groups. Therefore, the total built-in electric fields of the external Janus monolayers are higher than those of the internal Janus materials due to the higher electronegativity differences between *Q*′ and *Q* than between *A* and *A*′ (and, hence, higher charge transfer differences), resulting in larger electric field differences between the *AQ* and *A*′*Q*′ groups. Full-Janus has a similar trend as a result of the different chalcogens on the surfaces.

The dipole moments obtained from our simulations allow us to further quantify these polarization effects. Polarization, calculated as the dipole moment perpendicular to the monolayer plane per unit area, is greater than 1.8 me/Å for structures with mixed chalcogens (external and full-Janus) and less than 0.51 me/Å for internal-Janus structures. These values agree with the Bader charge analysis presented in the previous subsection, as mixed chalcogens result in higher polarizations due to their localization at the surface, in contrast to mixed Ge and Sn in the internal region.

#### 3.3.4 Energy Shifts

**Figure 6:**
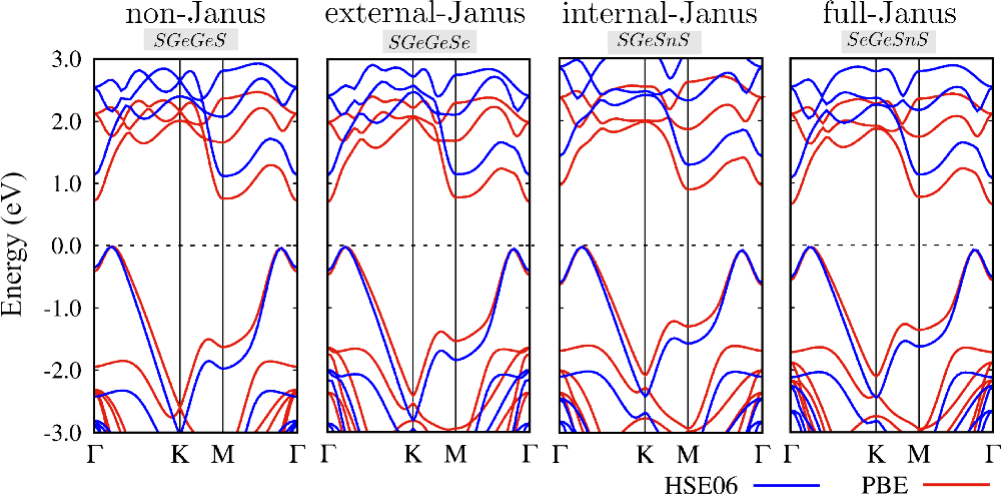
Electronic band structures calculated with the PBE and HSE06 functional. The Fermi energy is set to zero energy. The remaining electronic band structures can be accessed in the SI

Figure 6 illustrates the electronic band structures computed using both the PBE and HSE06 functionals for non-Janus and Janus structures. The HSE06 functional is expected to yield more precise values as a result of its reduced self-interaction errors. All of the band structures exhibit pronounced electron–hole asymmetry and feature indirect band gaps. The maximum valence band (VBM) is located along the Γ-*K* direction, whereas the minimum conduction band (CBM) is located at the Γ-point.

We also consider the effects of spin–orbit coupling due to the presence of tin atoms, which induces a band gap opening around 0.10 eV in other 2D materials, such as stanene.^79^ Comparing the band structures at the calculation levels of PBE and PBE+SOC, it is possible to note only small splittings in some conduction band states with higher energies in the Γ–*K* direction. The valence band remains unaltered under inclusion of spin–orbit coupling. Thus, it is safe to neglect the spin–orbit coupling in these materials.

Using the hybrid HSE06 functional, the assessment of the band structure addresses the commonly observed underestimation of the band gap that is inherent in the PBE functional.^84−87^ Within the energy range of interest (−3 to 3 eV), there is a nearly uniform upward shift of the conduction band. However, the valence band changes its shape and the dispersion increases. Self-interaction errors are more prominent for states with localized wave functions than for those with delocalized ones, and the flat regions of electronic band structures exhibit states that are more localized compared to the states in dispersive regions. That is, the flat region present in the electronic bands with energies close to −1.5 eV decreases in energy compared to those calculated at the PBE level, while a dispersive character is observed at the top of the valence bands, which remains almost unchanged. Therefore, a more precise characterization of the electronic states of Janus *P*3*m*1 monochalcogenides requires the use of functionals with lower self-interaction errors, such as the HSE06 used here.

#### 3.3.5 Effect in the Band Alignment

**Figure 7:**
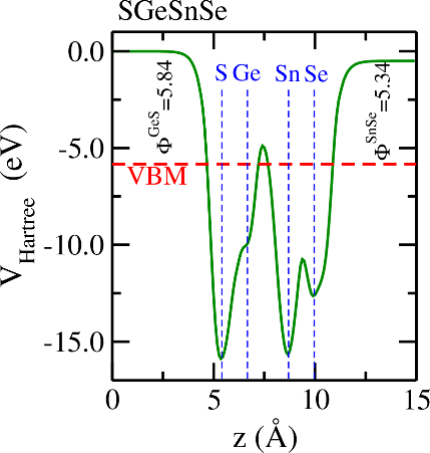
Average Hartree potential for SGeSnSe in the direction perpendicular to monolayer surface (*z*) calculated with the hybrid HSE06 functional. The dashed red line indicates the Fermi level and the blue dashed lines indicate the position of atomic layer of each specie. It is also shown the work functions for the GeS (Φ^GeS^) and SnSe (Φ^SnSe^) surfaces in units of eV. Differences between these values arise from the point-inversion-symmetry breaking. The average Hartree portentials for all the other materials can be accessed in the SI.

To calculate the properties of electron emission, we compute the work function,^88−90^ which can be defined as Φ^*QA*^ = *E*_*V*_^*QA*^ – *E*_F_, where *E*_*V*_^*QA*^ is the plateau in the average Hartree potential far a few Å from the surface with termination *QA*, and *E*_F_ is the Fermi energy. It is well-known that 2D Janus structures have different work functions for opposed surfaces due to built-in electric fields induced by point-inversion symmetry breaking.^83^Figure 7 shows an illustrative example for the lowest formation energy SGeSnSe full-Janus structure, which has higher values for the work function on the GeS side than on the SnSe one. All non-Janus structures have the same work function value for both sides, and their values are presented in the SI alongside the data of all the other Janus structures.

**Figure 8:**
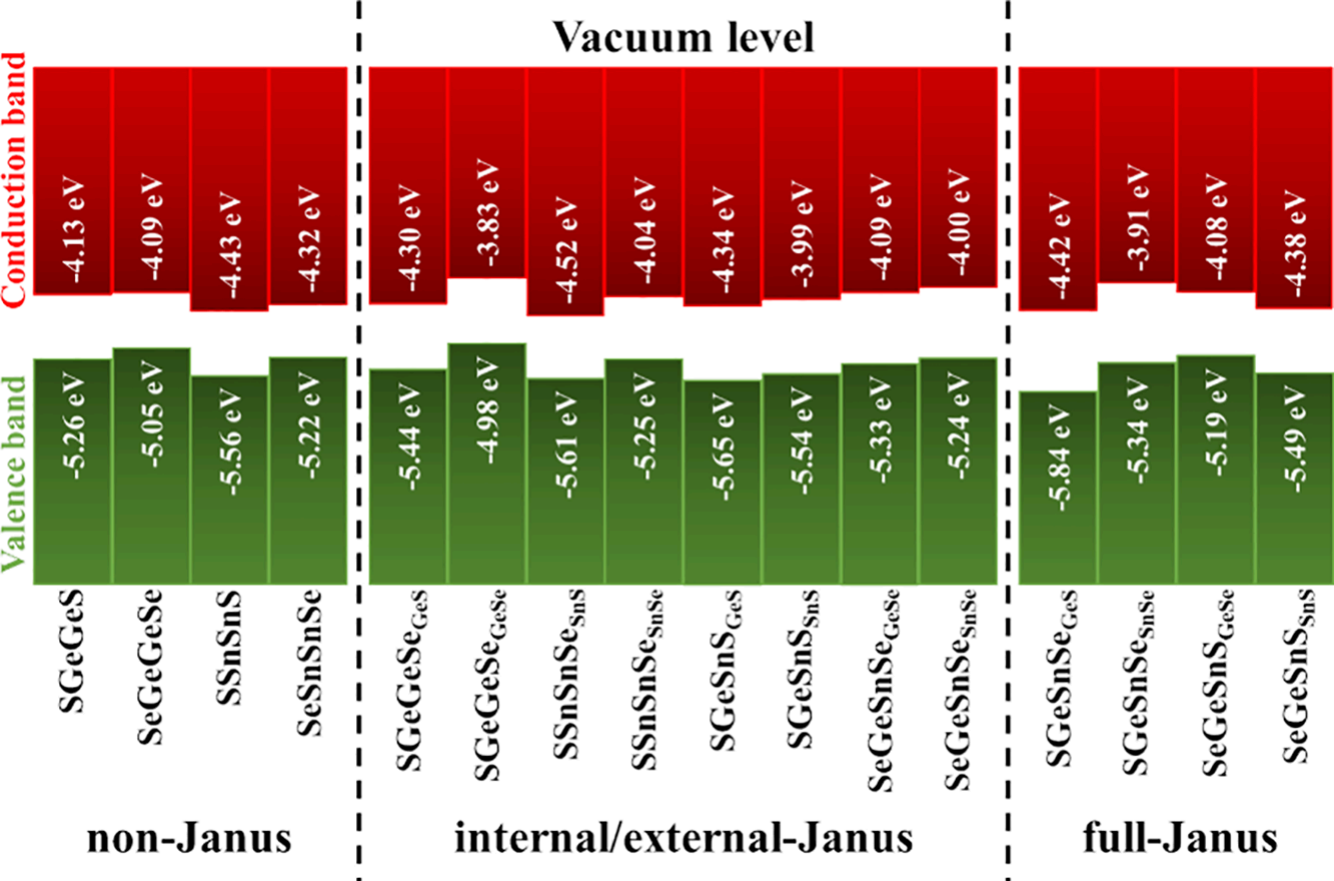
Band alignment for free-standing non-Janus and Janus compounds calculated with the hybrid HSE06 functional. For each Janus compound, the alignment of VBM and CBM with the vacuum levels *QA* and *A*′*Q*′ indicates in the subscript of each compound. Non-Janus compounds do not show different alignments.

Figure 8 depicts the band alignment for all 2D non-Janus and Janus structures considering *E*_*V*_^*QA*^ for both surfaces. It is worth noting that SGeGeSe and SGeSnSe present band edges differing by more than 0.5 eV from one side to the other. Moreover, in the limit of weak interactions, these band alignments indicate the formation of only type I and type II heterostructures (not type III) according to Anderson’s rule.^91^ These particular band alignments demonstrate potential for optical applications;^92^ however, interlayer interactions can easily change band alignments due to charge transfers and/or electrostatic interactions.^93^

### 3.4 Excitonic and Optical Properties

**Figure 9:**
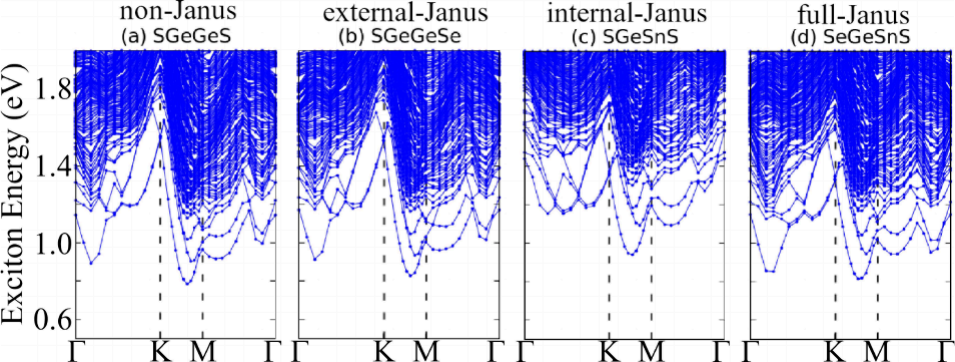
Exciton band structure for (a) SGeGeS, (b) SGeGeSe, (c) SGeSnS, and (d) SeGeSnS calculated with the DFT-MLWF-TB+BSE framework. The exciton band structures for the remaining chalcogenides are comprehensively detailed in the Supporting Information.

From the exciton band structure, in Figure 9, we observe that the exciton ground state (*Ex*_gs_) of the investigated systems are indirect, independent of the Janus group and the chemical composition, a fact that could be directly correlated with the indirect band gap of these semiconductors. For the non-Janus group, the *Ex*_gs_ is located between Γ–*K*, being the only exception SGeGeS where the ground state is between *K*–*M*, for the external/internal and full-Janus, our excitonic ground states are between *K*–*M*, except for the external SSnSnSe Janus, which is located between Γ–*K*.

In this work, we define the exciton binding energy (*Ex*_b_), listed in Table 2, as the difference between the fundamental band gap (*E*_g_) and the exciton ground state energy (*Ex*_gs_). These systems show *Ex*_b_ in the range of 0.20 to 0.37 eV, if the entire set is considered, with the lowest value for SeGeGeSe and the highest value for SGeSnS, showing how strong the quasiparticle effect is in these 2D systems. For the non-Janus group, *Ex*_b_ is in the range of 0.20 to 0.36 eV, with the lowest value for SeGeGeSe and the highest value for SGeGeS. Among the internal/external-Janus and full-Janus compounds, *Ex*_b_ lies in the range of 0.25 to 0.37 eV for the first group and 0.29 to 0.36 eV for the latter. These results show a higher dependence of *Ex*_b_ on the chemical composition of the monolayer, especially chalcogenides, where the highest (lowest) binding energies are found for S (Se), we also observe that the binding energies are higher in Janus compounds, suggesting that dipoles play an important role in the enhancement of exciton binding energy.

**Table 2:** MLWF-TB+BSE Calculated Excitonic Properties^a^

system	*E*_g_ (eV)	*E*_g_^*d*^ (eV)	*Ex*_gs_ (eV)	*Ex*_gs_^*d*^ (eV)	*Ex*_b_ (eV)
SGeGeS	1.13	1.49	0.77	1.14	0.36
SeGeGeSe	0.94	1.32	0.74	1.01	0.20
SSnSnS	1.13	1.60	0.84	1.20	0.29
SeSnSnSe	0.90	1.32	0.68	0.98	0.22
SGeGeSe	1.14	1.54	0.83	1.17	0.31
SSnSnSe	1.08	1.55	0.83	1.16	0.25
SGeSnS	1.31	1.92	0.94	1.44	0.37
SeGeSnSe	1.23	1.66	0.89	1.24	0.34
SGeSnSe	1.41	1.92	1.05	1.45	0.36
SeGeSnS	1.10	1.57	0.81	1.15	0.29

a Fundamental band gap (*E*_g_), direct band gap (*E*_g_^*d*^), exciton ground state (*Ex*_gs_), direct exciton ground state (*Ex*_gs_^*d*^), and exciton binding energy (*Ex*_b_), obtained from *E*_g_ – *Ex*_gs_. All direct excitons ground states are bright. The horizontal lines in the table separate classes of materials following the order non-Janus → external → internal → full-Janus.

**Figure 10:**
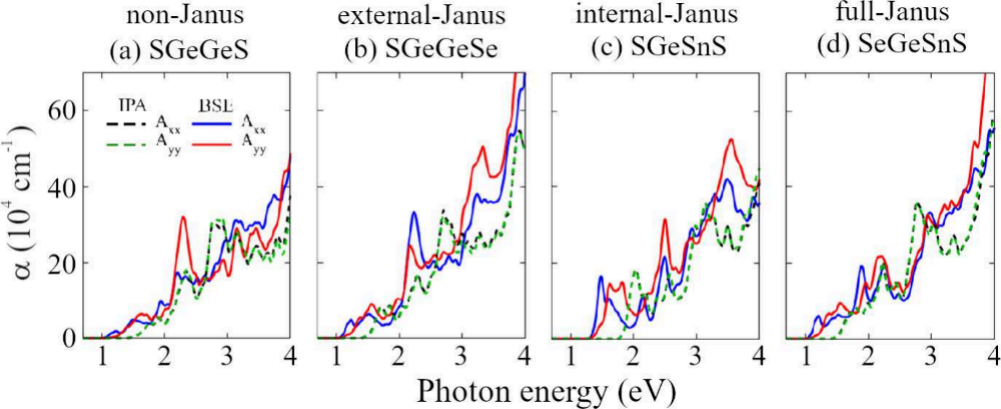
Absorption coefficient (α) for (a) SGeGeS, (b) SGeGeSe, (c) SGeSnS, and (d) SeGeSnS, considering linear light polarization along *x̂* and *ŷ* according to BSE (solid lines) and IPA (dashed lines). The absorption coefficient for all remaining systems is summarized in the SI.

In Figure 10 we show the absorption coefficient obtained at the BSE and IPA levels, considering a linear light polarization in the *x* and *y* directions, for one system of each group, complete data can be found in SI, Section 8. In the IPA level of theory, the spectrum is exactly the same regardless of the light polarization, which shows an isotropic behavior for light excitation; however, when excitonic effects are considered, the absorption peaks have different behavior for each polarization, which indicates a linear dichroism due to quasiparticle effects. The optical band gap is slightly different for each polarization, being smaller in the *x* direction (direct exciton ground state), indicating the possibility of using polarization filters to tune the band gap, through a linear combination of *x* and *y* light polarization.

### 3.5 Interaction with Light

The interaction of the selected monolayers with light was examined through the analysis of the power conversion efficiency (PCE) across three distinct theoretical frameworks: (*i*) at the Shockley–Queisser limit (PCE^SQ^), which considers that all photons with energy higher than the optical band gap are absorbed; (*ii*) modeling the absorbance as a Heaviside function, SLME (PCE^SLME^), which considers the total absorption coefficient (defined as the sum of *A*_*xx*_ and *A*_*yy*_ components) and layer thickness to model the absorbance; it also considers the recombination fraction, which reduces the PCE value for systems with indirect excitons (BSE) or indirect band gaps (IPA); (*iii*) we also consider PCE with maximum absorbance (PCE_*max*_^SLME^), as light trapping techniques can be used as a mechanism to improve the absorbance to values closer to 100%,^94^ evincing the maximum solar harvesting potential of these monolayers in a maximally optimized absorption situation. The limitations of this method arise from the assumptions of SQ^62^ and from the approach to the recombination factor.^30^Table 3 shows the calculated PCE for each of these levels.

**Table 3:** Maximum Achieved PCE at the IPA and BSE Levels, PCE^SLME^ (%), Power Conversion Efficiency Obtained by SLME Considering That 100% of Photon Absorbance Starts from the Direct Band Gap, PCE_max_^SLME^ (%) and Obtained in the Shockley–Queisser Limit (Considering Direct Band Gap), PCE^SQ^ (%), Calculated with *T* = 298.15 K^a^

	IPA			BSE		
system	PCE^SLME^	PCE_max_^SLME^	PCE^SQ^	PCE^SLME^	PCE_max_^SLME^	PCE^SQ^
SGeGeS	0.67	20.99	31.35	0.54	17.72	32.36
SeGeGeSe	0.82	19.44	32.61	0.75	18.22	30.34
SSnSnS	0.65	18.33	28.88	0.70	18.63	32.29
SeSnSnSe	0.75	18.19	32.55	0.67	16.04	30.14
SGeGeSe	0.82	20.26	30.93	0.72	18.81	32.37
SSnSnSe	0.73	18.43	30.77	0.67	18.93	32.37
SGeSnS	0.69	14.40	24.21	0.71	16.98	32.06
SeGeSnSe	0.99	19.04	29.06	0.86	19.24	32.07
SGeSnSe	0.88	15.97	24.21	1.00	19.88	31.89
SeGeSnS	0.80	18.27	30.42	0.72	18.67	32.37

a The horizontal lines in the table separate classes of materials following the order non-Janus → external → internal → full-Janus.

First, we analyze the PCE^SLME^, keeping in mind that the layer thickness on an atomic scale, typical of 2D materials, limits light absorption. We obtained values below 1% for all investigated materials. For comparison purposes, we contrast these values with experimentally measured PCEs, and note that despite the fact that specific materials can surpass 30%,^95^ the 2D monolayers have typical values not exceeding 2%.^96−98^ Thus, the values presented in Table 3 fall in a reasonable range for 2D materials.

Our simulations also demonstrate average PCE^SLME^ values for Janus materials slightly exceeding those for non-Janus materials by 13% and 17% in the IPA and BSE approaches, respectively, due to the larger band gap for Janus materials resulting from their point-inversion symmetry breaking. In fact, the PCE^SLME^ calculated from the BSE level falls in the range between 0.54% to 0.75% for non-Janus materials and 0.67% to 1.00% for Janus materials (that is, the maximum value among Janus structures is almost twice the minimum value for non-Janus ones), demonstrating the promising role of Janus structures in improving the efficiency of light conversion. Furthermore, the reduction in the band gap resulting from bound excitons versus the particle-independent approach increases PCE only for SSnSnS, SGeSnS, and SGeSnSe. For these three particular materials, the direct band gap in the IPA approach exceeds the maximum efficiency band gap value, and then the excitonic effects cause an additional band gap decrease, resulting in larger PCE values.

Table 3 also presents PCE_max_^SLME^ considering maximal light absorption that can be reached through device design.^94^ The average PCE_max_^SLME^ increase from non-Janus to Janus structures significantly decreases to only 1% within the BSE approach, whereas the IPA approach even predicts lower PCE_max_^SLME^ for Janus structures against non-Janus ones. Furthermore, the highest PCE_max_^SLME^ in the BSE approach still occurs for Janus SGeSnSe and is around 1% higher than its maximum value for non-Janus materials. Thus, it is worth stressing that the differences between Janus and non-Janus structures are most pronounced without maximizing light absorption.

Table 3 further presents PCE^SQ^, which is exclusively related to the band gap values. Again, there are only slight differences between Janus and non-Janus structures, corroborating the previous assertion that light absorption maximization reduces the differences among Janus and non-Janus structures. Although the IPA approach results in PCE^SQ^ exceeding 24%, the inclusion of excitonic effects through BSE increases PCE^SQ^ to over 30% for all materials. These results demonstrate that all investigated monolayers have band gaps suitable for energy harvesting and that excitonic effects increase device efficiency because of the influence of point-inversion symmetry breaking on the band gap values. However, the limited thickness results in low light absorption, which reduces efficiency.

## 4 Insights

In this study, we systematically quantified the effects of point-inversion symmetry breaks in group-IV monochalcogenides by contrasting the optoelectronic properties between non-Janus and Janus structures with similar hexagonal crystal phases and analogous compositions. Anticipating our results, our findings unequivocally demonstrate that Janus structures enhance, albeit modestly, the interaction with sunlight compared to their non-Janus counterparts, attributed to the influence of excitonic effects.

Through property analysis, we identified a strong influence of the core region. The lattice parameter (*a*) increased by at least 0.14 Å when changing one of the core layers from Ge to Sn. The same behavior is observed when the elastic properties are analyzed, with a variation of 15N m^–1^ when the core region is changed, demonstrating that this region governs the hardness of the materials. Band gaps were also primarily affected by the core region, adding at least 0.18 eV when transitioning from the non-Janus to the internal-Janus.

Excitons play an important role in the optical band gap of these group-IV monochalcogenide materials. The exciton binding energy *Ex*_b_ is greater than 21% of the values obtained for the fundamental band gap *E*_g_ for all compounds. Therefore, even though Janus structures lead to an increase in the band gap beyond the maximum efficiency threshold of the SQ (1.3 eV),^62^ the excitonic effect reduces it to near the optimal value. We used the ideal theoretical PCE^SQ^ as a measure of the material/sunlight interaction, and it increases by up to 8% when considering excitonic effects. Moreover, the absorption coefficient was also affected by the addition of quasiparticle effects. It is expected that there is no anisotropy in hexagonal structures; however, the light polarization in the *x̂* and *ŷ* directions presented anisotropy caused by excitons.

Thus, in the particular case of *P*3*m*1 and *P*3̅*m*1 *QAA*′*Q*′ monochalcogenides, the optoelectronic properties were mostly dominated by excitonic effects. However, the construction of Janus structures generates a band gap increase due to the point-inversion symmetry breaking necessary to maximize the material/sunlight interaction.

## 5 Conclusions

Using a combination of first-principles calculations based on density functional theory and solutions of the Bethe–Salpeter equation, we explored the physical-chemical properties of the following compounds: 2D SGeGeSe, SSnSnSe, SGeSnS, SeGeSnSe, SGeSnSe, and SeGeSnS *P*3*m*1 monochalcogenides in Janus structures and contrasted the results with those obtained for the non-Janus SGeGeS, SeGeGeSe, SSnSnS, and SeSnSnSe *P*3̅*m*1 analogous materials. The widely used GGA (PBE) exchange-correlation energy functional was employed to generate the structural, energetic, and elastic properties. However, the hybrid HSE06 functional was employed to improve the description of the electronic structure and, hence, an accurate parametrization for the tight-binding Hamiltonian that informs the Bethe–Salpeter equation.

The selected Janus materials disrupt the point-inversion symmetry through internal mechanisms (through the incorporation of group-IV elements Ge and Sn) and external mechanisms (through the utilization of distinct chalcogens (S and Se) as well as their combinations throughout the Janus structure. Due to the fact that Ge and S have radii shorter than Sn and Se, we could verify a good correlation between the lattice parameter (*a*) and the atomic radii. Moreover, the calculated phonon band structures show no imaginary frequencies for all acoustic branches, indicating that all structures are dynamically stable. To check the mechanical stability, we considered Maździarz’s stability criterion and also calculated the angular dependence of Young’s modulus and Poisson’s ratio, which presented a perfect circular shape, confirming the isotropic elastic properties of hexagonal 2D materials. The energetic stability was analyzed through the formation enthalpy and formation energy, indicating robust stability for all Janus materials except for SGeSnS and SGeSnSe which have formation energies 9.1 and 32.1 meV/u.c., respectively, higher than the already synthesized MoSSe Janus material, thus, being challenging to grow. In particular, SeGeSnS full-Janus monolayer has a formation energy of −0.9 meV/u.c., which demonstrates competitive energy compared to non-Janus materials, highlighting its significance in the field of Janus materials. Thus, we meticulously confirm the stability of all materials.

For electronic properties, all compounds have indirect band gaps in the range of 0.7 up to 1.2 eV. We were able to indicate that our systems possess ionic bonds through a Bader charge analysis, and we could also identify symmetry breaking through both Bader charge and work function evaluations. Moreover, the density of states showed clear signatures of Janus point symmetry breaking in the energy region close to the VBM and CBM.

The exciton binding energy (*Ex*_b_) varies in a range of 0.20 to 0.36 eV for non-Janus structures and in a range of 0.25 to 0.37 eV for Janus structures, with the lowest binding energy observed in SSnSnSe and the highest in SGeSnS. The excitonic binding energy is highly dependent on the composition of the monolayer chalcogenide, being higher with S. We also observe that Janus compounds tend to have higher *Ex*_b_, a result that is correlated with the presence of dipoles due to the breaking of the point-inverstion symmetry in Janus structures. When excitonic effects were considered in the absorption coefficients, linear dichroism owing to quasi-particle effects is observed. These systems also show a solar harvesting efficiency of around 16% when light trapping techniques are applied to the solar harvesting device, suggesting enhanced material/sunlight interactions due to excitonic effects.

Our results demonstrate that excitonic effects strongly affect the optoelectronic properties (band gap and absorption coefficient) more than the point-inversion symmetry break inherent in Janus structures. However, both features are necessary to enhance the materials/sunlight interaction.

## Supporting Information Available

Essential technical details that complement and support the reproduction of the published simulations, e.g., specific simulation parameters, optimized geometries, total energy values, additional local DOS figures, spin–orbit coupling tests, additional electronic band structures, data used for calculating the band alignment, technical details for calculating the Wannier functions, and additional plots for the excitonic properties (PDF)
