# Supplementary material for: Tuning Excitonic Properties of Monochalcogenides via Design of Janus Structures
Source: J Phys Chem C Nanomater Interfaces. 2024 Jul 12;128(29):12164–77. doi: 10.1021/acs.jpcc.4c01813 (PMC11284856; doi:10.1021/acs.jpcc.4c01813)
Supplement: Supplementary file 1 — jp4c01813_si_001.pdf [file jp4c01813_si_001.pdf]

# Supporting Information: Tuning Excitonic Properties of Monochalcogenides via Design of Janus Structures

Mateus B. P. Querne,*,† Alexandre C. Dias,*,‡ Anderson Janotti,¶ Juarez L. F. Da Silva,*,§ and Matheus P. Lima*,†

†*Department of Physics, Federal University of São Carlos,* 13565-905*, São Carlos, São Paulo, Brazil*

‡*University of Brasília, Institute of Physics and International Center of Physics, Brasília* 70919-970*, DF, Brazil*

¶*Department of Materials Science and Engineering, University of Delaware, Newark, Delaware* 19716*, USA*

§*São Carlos Institute of Chemistry, University of São Paulo, P.O. Box* 780*,* 13560-970*, São Carlos, São Paulo, Brazil*

E-mail: mateusbpq@gmail.com; alexandre.dias@unb.br; juarez_dasilva@iqsc.usp.br; mplima@df.ufscar.br

## Contents

**S1 Introduction** **S2**

**S2 Selected PAW Projectors: Technical Details** **S3**

**S3 Optimized Geometries** **S3**

**S4 Phonon band structures** **S7**

**S5 Energetic and Structural Reference Values** **S8**

S5.1 Free Atoms . . . . . . . . . . . . . . . . . . . . . . . . . . . . . . . . . . S8

S5.2 Monolayers . . . . . . . . . . . . . . . . . . . . . . . . . . . . . . . . . . S8

S5.2.1 Janus Lattice Parameter Estimate . . . . . . . . . . . . . . . . . . . . . S9

**S6 Electronic Properties** **S9**

S6.1 Density of States . . . . . . . . . . . . . . . . . . . . . . . . . . . . . . S9

S6.2 Spin-Orbit Coupling Effects . . . . . . . . . . . . . . . . . . . . . . . . . . S16

S6.3 PBE *.vs.* HSE06 electronic band structures . . . . . . . . . . . . . . . . . . . . . S18

S6.4 Workfunction . . . . . . . . . . . . . . . . . . . . . . . . . . . . . . . . . S19

**S7 Wannierization** **S20**

**S8 Excitonic and Optical Properties** **S23**

**References** **S33**

# S1 Introduction

This Supplementary Information provides essential technical details that complement and support the reproduction of the published simulations. It encompasses specific simulation parameters, optimized geometries, total energy values, relevant published results, local density of states, spin-orbit coupling tests, electronic band structures, data utilized for calculating the band alignment, methodology details for calculating the Wannier functions, and visual plots illustrating the excitonic properties. These supplementary materials are intended to facilitate a comprehensive understanding of the research findings and aid fellow researchers in replicating and extending the reported results.

## S2 Selected PAW Projectors: Technical Details

Table S1: Key specifications of selected PAW projectors, including Species, PAW-PBE projector name (Title), electronic valence configuration (Valence), number of valence electrons ($Z_{val}$), and maximum recommended cutoff energy (ENMAX).

| Species | Title | Valence | $Z_{val}$ | ENMAX (eV) |
|---|---|---|---|---|
| Ge | Ge_GW 04Okt2005 | $4s^2 4p^2$ | 4 | 173.807 |
| Sn | Sn_d_GW 15Mar2013 | $4d^{10} 5s^2 5p^2$ | 14 | 260.066 |
| S | S_GW 19Mar2012 | $3s^2 3p^4$ | 6 | 258.689 |
| Se | Se_GW 20Mar2012 | $4s^2 4p^4$ | 6 | 211.555 |

## S3 Optimized Geometries

Optimized geometries in the VASP POSCAR format.

```
SGeGeS non-Janus
1.00000000000000
3.1641797803846803 -1.8268475805853308 0.0000000000000000
3.1641754033071985 1.8268400054353073 0.0000000000000000
0.0000000000000000 0.0000000000000000 20.0000000000000000
Ge S
2 2
Direct
0.3333209530707080 0.3333466577409062 0.5786287668403658
0.6666587955413928 0.6666773185451441 0.6831113407124833
0.0000000536222007 0.0000168833903518 0.5160622254930468
0.0000007107391281 0.0000164866102148 0.7456773519541022
```

```
SeGeGeSe non-Janus
1.00000000000000
3.2975038417618161 -1.9038197907544292 0.0000000000000000
3.2974988883175884 1.9038112175821016 0.0000000000000000
0.0000000000000000 0.0000000000000000 20.0000000000000000
Ge Se
2 2
Direct
0.3333247138445360 0.3333504185148028 0.5802110061190477
0.6666609227004759 0.6666794457042533 0.6814934434806211
0.9999977455388800 0.0000145753069435 0.5124341088399614
0.9999971308895379 0.0000129067606176 0.7493411265603678
```

```
SSnSnS non-Janus
1.00000000000000
3.4207572651404381 -1.9749836859725347 0.0000000000000000
3.4207518219556263 1.9749742647738917 0.0000000000000000
0.0000000000000000 0.0000000000000000 20.0000000000000000
Sn S
2 2
Direct
0.3333228613174958 0.3333485659880731 0.5683623368483881
0.6666589224214372 0.6666774454251547 0.6933760098169166
0.9999986574228208 0.0000154871907501 0.5024178986536040
0.0000000718116761 0.0000158476826321 0.7593234396810823
```

```
SeSnSnSe non-janus
1.00000000000000
3.5433189609690081 -2.0457336969984081 0.0000000000000000
3.5433180840942091 2.0457321920195288 0.0000000000000000
0.0000000000000000 0.0000000000000000 20.0000000000000000
Sn Se
2 2
Direct
0.3333273430158729 0.3333469911981148 0.5708261134990493
0.6666628430577186 0.6666818186957767 0.6909174322688845
0.9999941962450152 0.0000145920731282 0.4992674953615721
0.9999961306548091 0.0000139443196044 0.7624686438704922
```

```
SGeGeSe external-Janus
1.00000000000000
3.2338571582123365 -1.8670737696240314 0.0000000000000000
3.2338524758030407 1.8670656657700990 0.0000000000000000
0.0000000000000000 0.0000000000000000 20.0000000000000000
Ge S Se
2 1 1
Direct
0.3333261887397947 0.3333518934099975 0.5771130259737983
0.6666624982863073 0.6666810212900608 0.6800326088389809
0.9999944596653434 0.0000112894334931 0.5167846281458466
0.9999973662819843 0.0000131421530658 0.7495494220413724
```

```
SGeSnS internal-Janus
1.000000000000
3.297312023730 -1.903707711247 0.000000000000
```

```
3.297308194277 1.903701076369 0.000000000000
0.000000000000 0.000000000000 20.000000000000
Ge Sn S
1 1 2
Cartesian
2.198248959609 0.000049317724 11.414642695013
4.396447706815 0.000036212891 13.695368432595
3.297326717929 -1.903682718620 10.261000175905
3.297345714689 -1.903678601577 15.098582396487
```

```
SeGeSnSe internal-Janus
1.000000000000
3.420747536013 -1.974973673199 0.000000000000
3.420746817810 1.974971429392 0.000000000000
0.000000000000 0.000000000000 20.000000000000
Ge Sn Se
1 1 2
Cartesian
2.280530814535 0.000044352061 11.449955085651
4.561031220477 0.000034081626 13.662800388861
3.420772329520 -1.974935506464 10.180064329819
3.420784502362 -1.974940800081 15.176773945669
```

```
SSnSnSe external-Janus
1.000000000000
3.482986077612 -2.010904703372 0.000000000000
3.482983887699 2.010900904316 0.000000000000
0.000000000000 0.000000000000 20.000000000000
Sn S Se
2 1 1
Cartesian
2.322018840752 0.000039006692 11.341812404184
4.644015947502 0.000033232337 13.791121508094
3.483016206213 -2.010862239455 10.073035338688
3.483022989222 -2.010868701120 15.263624449034
```

```
SGeSnSe full-Janus
GeSnS2
1.00000000000000
3.3578552285819585 -1.9386625488419185 0.0000000000000000
3.3578483632544742 1.9386506556469283 0.0000000000000000
0.0000000000000000 0.0000000000000000 20.0000000000000000
Ge Sn S Se
```

```
1 1 1 1
Direct
0.3333280107012726 0.3333496513306713 0.5689030019704732
0.6666621293301489 0.6666806393754996 0.6819936025245754
0.9999929472567857 0.0000142259150167 0.5132827681907539
0.9999974256854927 0.0000128296655433 0.7593003123141883
```

```
SeGeSnS full-Janus
1.00000000000000
3.3633991095026636 -1.9418633276628319 0.0000000000000000
3.3633983677584940 1.9418620408006200 0.0000000000000000
0.0000000000000000 0.0000000000000000 20.0000000000000000
Ge Sn S Se
1 1 1 1
Direct
0.3333282840665319 0.3333491046001384 0.5740322928014925
0.6666616042581879 0.6666816895194074 0.6863141742949510
0.9999975257038756 0.0000126296287846 0.7544449897331751
0.9999930989451045 0.0000139225384004 0.5086882281703796
```

# S4 Phonon band structures

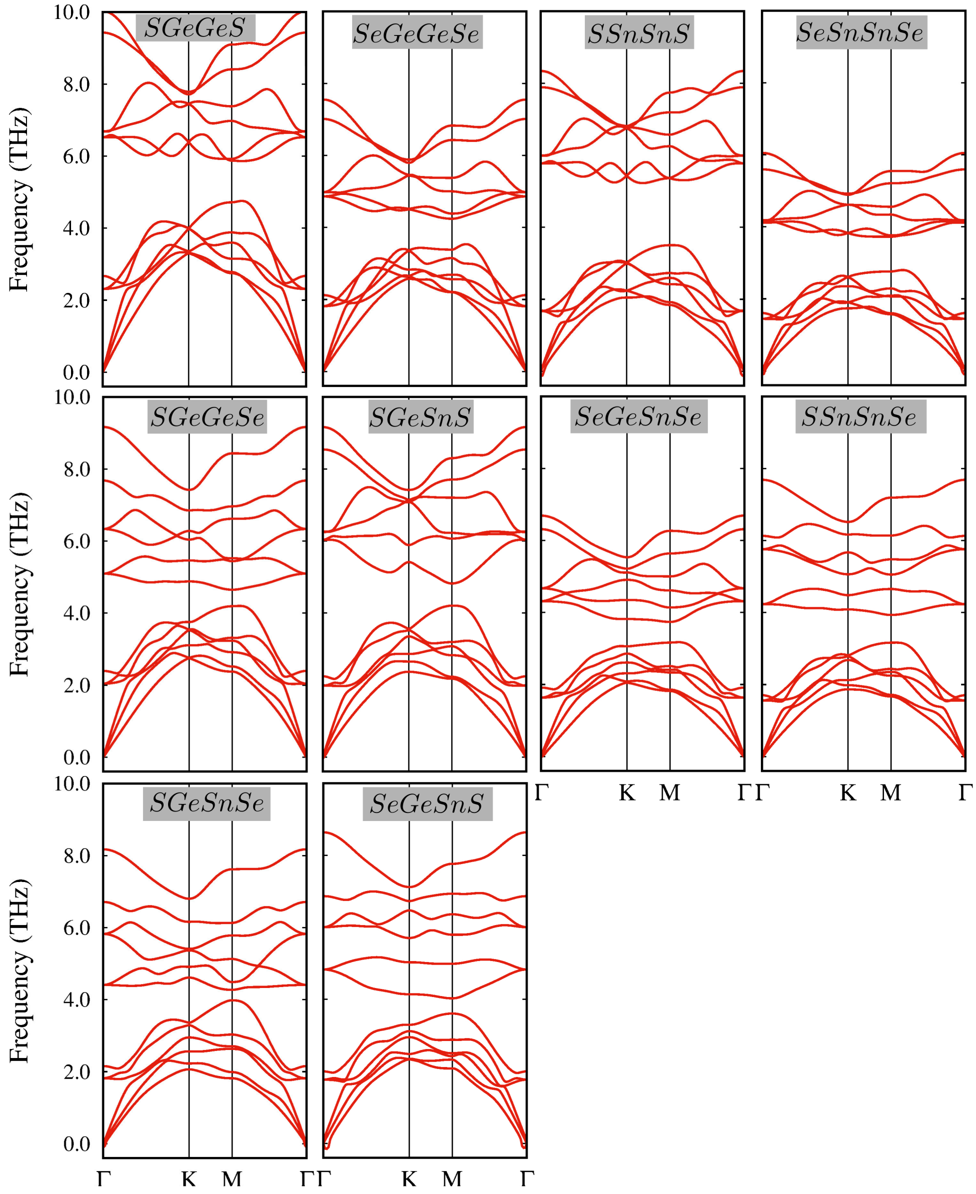


Figure S1: Phonon band structures calculated with PBE exchange-correlation functional with a 3×3×1 supercell.

# S5 Energetic and Structural Reference Values

This section presents information of the reference systems used in the evaluation of the energetic properties, such as (i) total energy of free atoms used for calculating the cohesive energy; (ii) total energies of non-Janus structures with $P\bar{3}m1$ space group used for calculating formation energies; and (iii) total energies of phosphorene-like (ph-like) Janus structures for evaluate relative energies.

## S5.1 Free Atoms

The free-atom calculations adopt an orthorhombic box of 20 Å×21 Å×22 Å to avoid undesirable symmetry constraints, we adopt a small Gaussian smearing parameter value ($\sigma$) to guarantees the absence of partial occupation of the atomic orbitals. Table S2 presents the total energies of free atoms that compose the investigated Janus systems. We also present the Gaussian smearing parameter ($\sigma$), the occupation of the highest-occupied-molecular-orbital, HOMO, ($\mathrm{OCC}_{\mathrm{HOMO}}$), and the HOMO eigenvalue.

Table S2: Free-atom calculations. Species, Gaussian smearing parameter ($\sigma$); HOMO eigenvalue ($E_{\mathrm{HOMO}}$), HOMO occupation ($\mathrm{OCC}_{\mathrm{HOMO}}$); Total energy ($E_{\mathrm{tot}}$).

| Species | $\sigma$ (eV) | $E_{\mathrm{HOMO}}$ (eV) | $\mathrm{OCC}_{\mathrm{HOMO}}$ | $E_{\mathrm{tot}}$ (eV) |
|---|---|---|---|---|
| Ge | 0.0001 | −4.35 | 1.00 | −0.775 568 |
| Sn | 0.0001 | −4.09 | 1.00 | −0.674 855 |
| S | 0.0001 | −6.80 | 1.00 | −1.054 679 |
| Se | 0.0001 | −6.34 | 1.00 | −0.884 376 |

## S5.2 Monolayers

Table S3 presents a comparison between our calculated lattice parameters and values reported in the literature, with the aim of validating our simulations. We conclude that our simulations agree with recent published data. The Atomic Simulation Environment (ASE) tool classifies the space group for each compound by adopting `symprec`=$1 \times 10^{-3}$ Å.

Table S3: Lattice parameter and total energy for free-standing monolayers, namely, Composition; classification as non-Janus, internal-Janus, external-Janus, or full-Janus; space group; lattice parameters ($a_0$); and total energy ($E_{\text{tot}}$). The $P\bar{3}m1$ spacial group contains inversion center symmetry, whereas $P3m1$ not.

| source | Composition | crystal phase | space group | $a_0$ (Å) | $E_{\text{tot}}$ (eV) |
|---|---|---|---|---|---|
| this work | SGeGeSe | external-Janus | $P3m1$ | 3.734 | −17.639 528 |
| this work | SGeSnS | internal-Janus | $P3m1$ | 3.807 | −17.798 777 |
| this work | SeGeSnSe | internal-Janus | $P3m1$ | 3.950 | −16.631 677 |
| this work | SSnSnSe | external-Janus | $P3m1$ | 4.022 | −16.832 100 |
| this work | SGeSnSe | full-Janus | $P3m1$ | 3.877 | −17.162 473 |
| this work | SGeSnSe | full-Janus | $P3m1$ | 3.884 | −17.231 727 |
| this work | SGeGeS | non-Janus | $P\bar{3}m1$ | 3.654 | −18.275 209 |
| Ref. [1] | SGeGeS | non-Janus | $P\bar{3}m1$ | 3.65 | - |
| this work | SeGeGeSe | non-Janus | $P\bar{3}m1$ | 3.808 | −17.048 757 |
| Ref. [1] | SeGeGeSe | non-Janus | $P\bar{3}m1$ | 3.80 | - |
| Ref. [2] | SeGeGeSe | non-Janus | $P\bar{3}m1$ | 3.808 | - |
| this work | SSnSnS | non-Janus | $P\bar{3}m1$ | 3.950 | −17.412 883 |
| Ref. [1] | SSnSnS | non-Janus | $P\bar{3}m1$ | 3.95 | - |
| this work | SeSnSnSe | non-Janus | $P\bar{3}m1$ | 4.091 | −16.273 866 |
| Ref. [1] | SeSnSnSe | non-Janus | $P\bar{3}m1$ | 4.09 | - |
| Ref. [2] | SeSnSnSe | non-Janus | $P\bar{3}m1$ | 4.089 | - |

#### S5.2.1 Janus Lattice Parameter Estimate

Table S4 contrast values for simulated lattice parameters of 2D Janus monolayers with chemical formula $QAA'Q'$ with average lattice parameters for monolayers with chemical formula $QAAQ$ and $Q'A'A'Q'$. The discrepancies are shorter than 0.01 Å, demonstrating that averages of non-Janus lattice patameters can be accuratelly used to determine lattice parameters for Janus monolayers.

# S6 Electronic Properties

## S6.1 Density of States

Figure S2 shows the density of states of all systems showing the contribution of each element, while Figures S3–S12 shows the contribution of each orbital in the density of states. All density of states were calculated with the PBE exchange-correlation energy functional.

Table S4: Janus lattice parameter estimate from non-Janus systems. $a_0^{QAA'Q'}$ is the optimized lattice parameter for Janus monolayers; and $a_0^{QAAQ}$ is the lattice parameter for non-Janus monolayers, both obtained from stress-tensor calculations with `ENCUT`=520 eV and a **k**-mesh of 9×9×1.

| system | $a_0^{QAA'Q'}$ (Å) | $1/2\left(a_0^{QAAQ}+a_0^{Q'A'A'Q'}\right)$ (Å) |
|---|---|---|
| SGeGeSe | 3.730 | 3.730 |
| SGeSnS | 3.810 | 3.800 |
| SeGeSnSe | 3.950 | 3.950 |
| SSnSnSe | 4.020 | 4.020 |
| SGeSnSe | 3.880 | 3.870 |
| SeGeSnS | 3.880 | 3.870 |

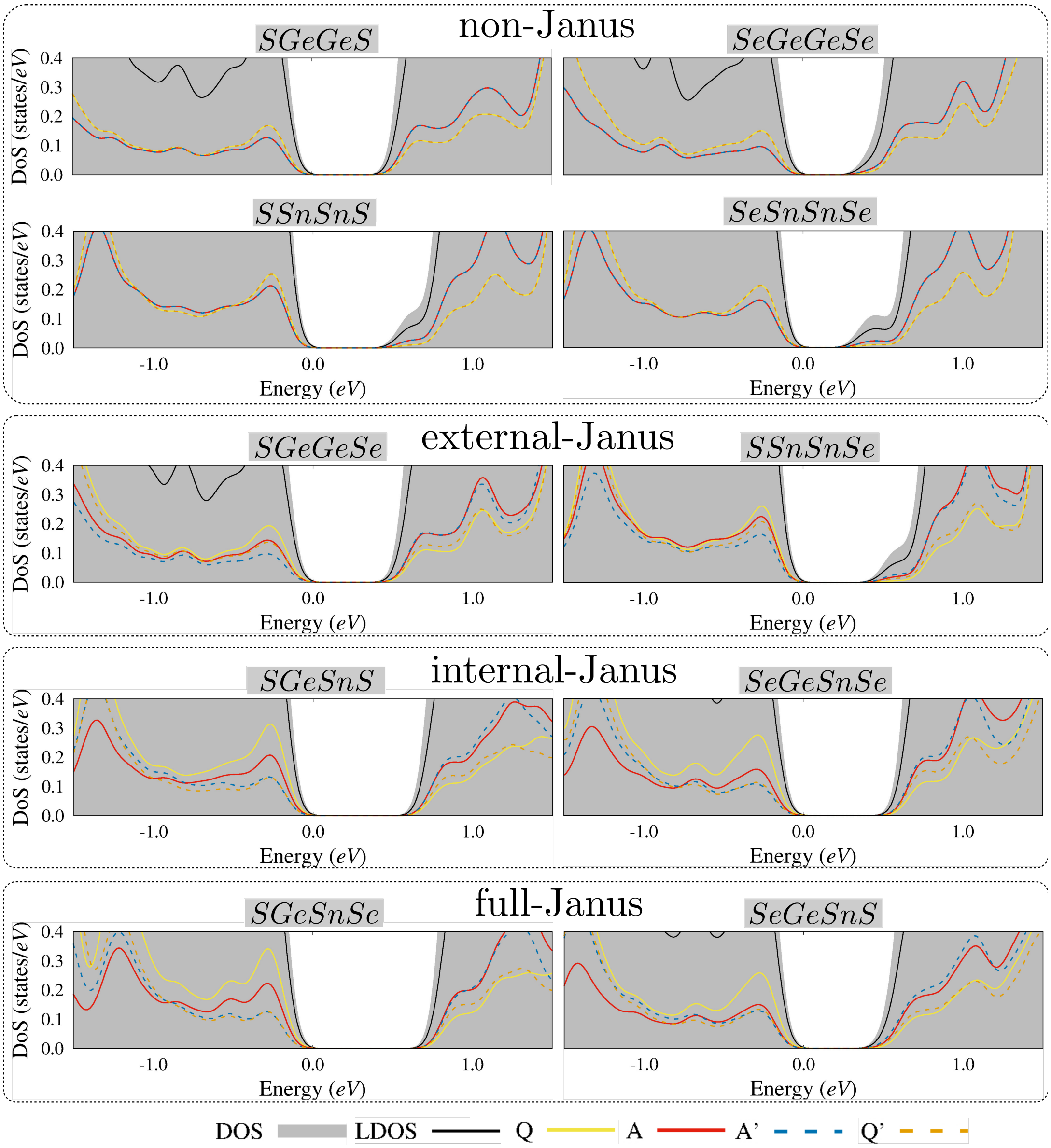


Figure S2: Density of states calculated with the PBE exchange-correlation energy functional. The Fermi level is set to zero.

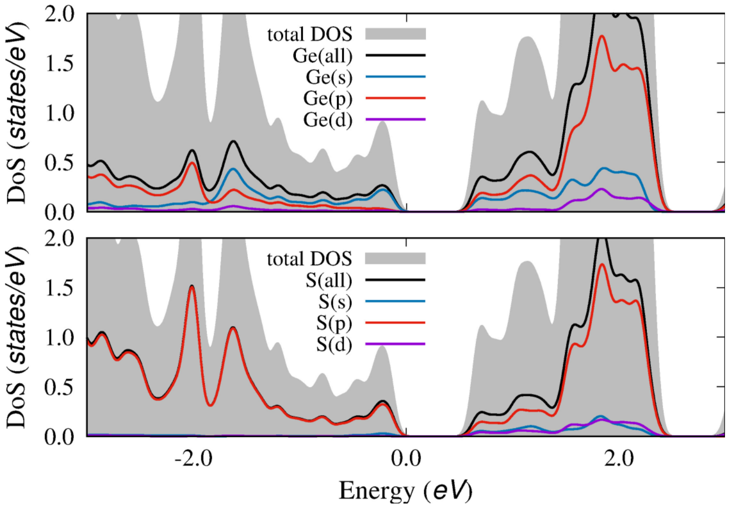


Figure S3: SGeGeS non-Janus: orbital resolved density of states calculated with the PBE exchange-correlation energy functional. The Fermi level is set to zero.

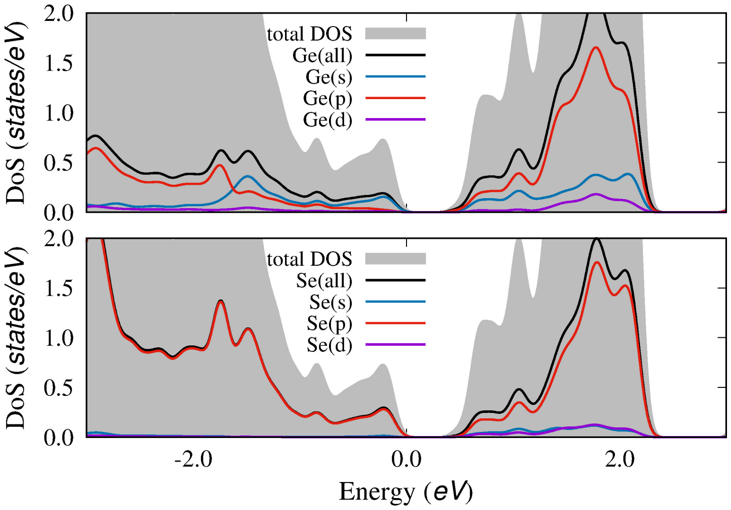


Figure S4: SeGeGeSe non-Janus: orbital resolved density of states calculated with the PBE exchange-correlation energy functional. The Fermi level is set to zero.

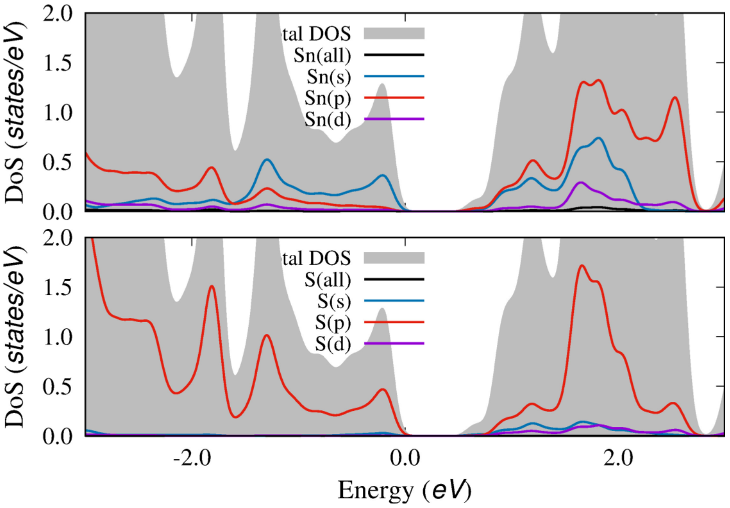


Figure S5: SSnSnS non-Janus: orbital resolved density of states calculated with the PBE exchange-correlation energy functional. The Fermi level is set to zero.

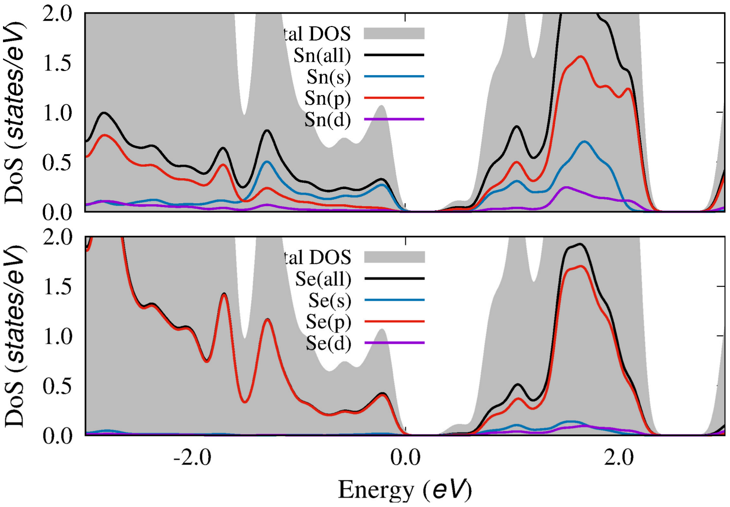


Figure S6: SeSnSnSe non-Janus: orbital resolved density of states calculated with the PBE exchange-correlation energy functional. The Fermi level is set to zero.

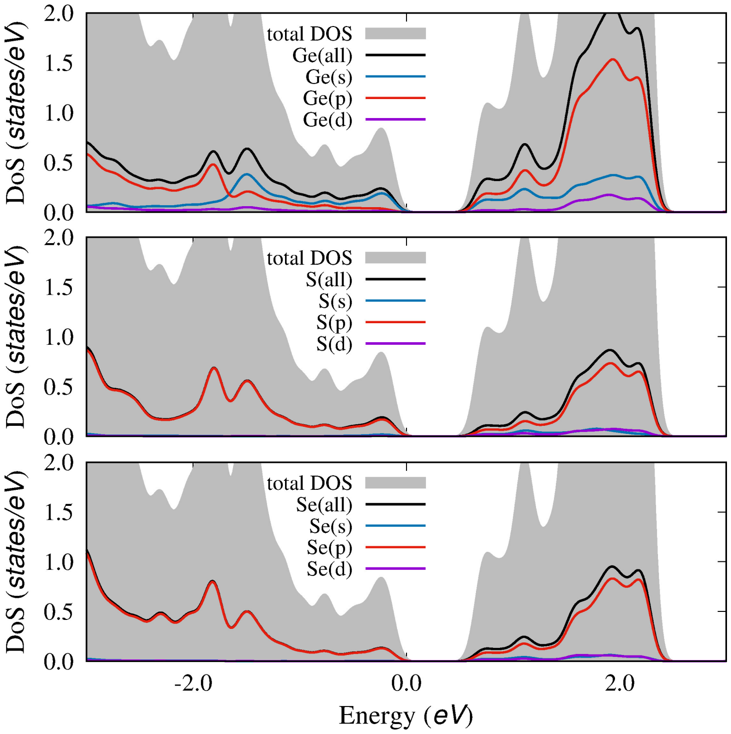


Figure S7: SGeGeSe external-Janus: orbital resolved density of states calculated with the PBE exchange-correlation energy functional. The Fermi level is set to zero.

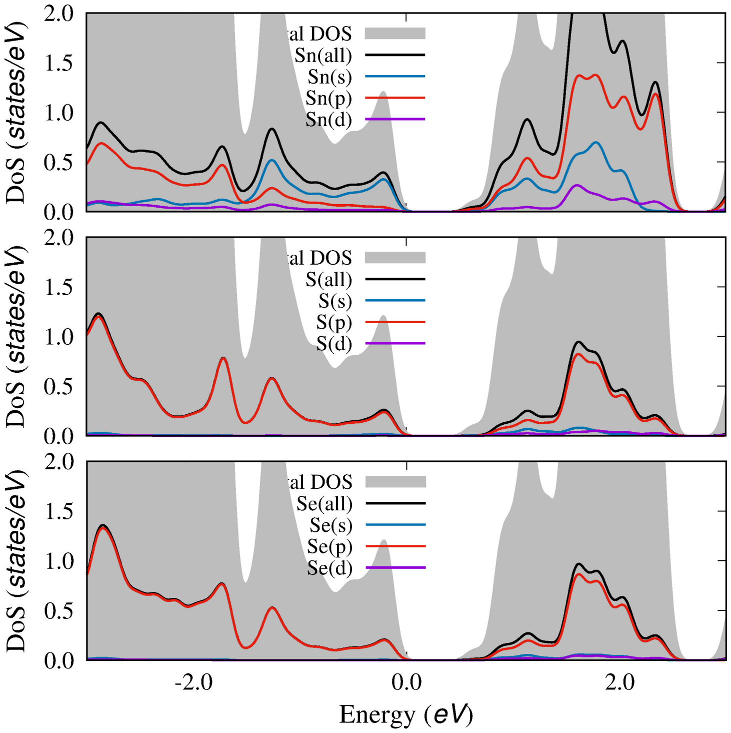


Figure S8: SSnSnSe external-Janus: orbital resolved density of states calculated with the PBE exchange-correlation energy functional. The Fermi level is set to zero.

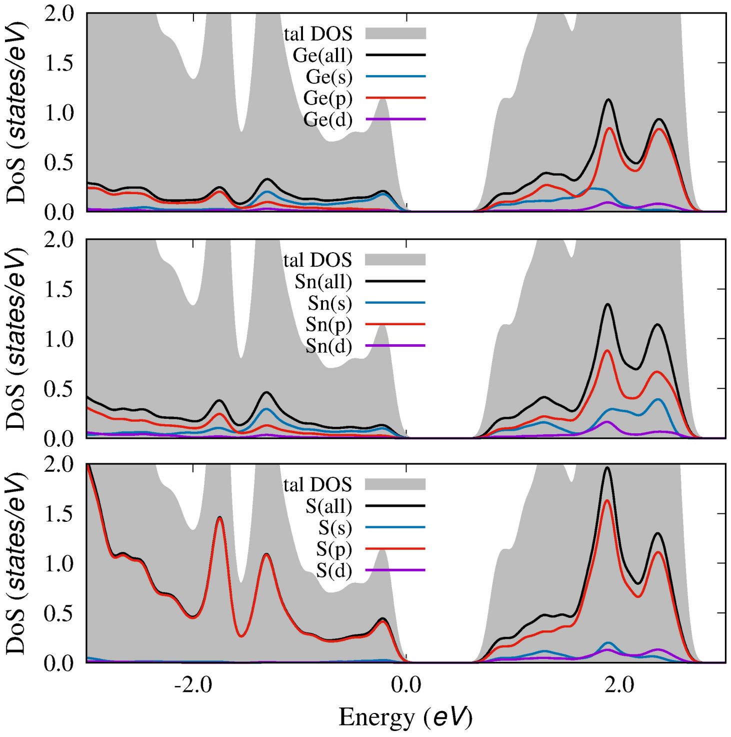


Figure S9: SGeSnS internal-Janus: orbital resolved density of states calculated with the PBE exchange-correlation energy functional. The Fermi level is set to zero.

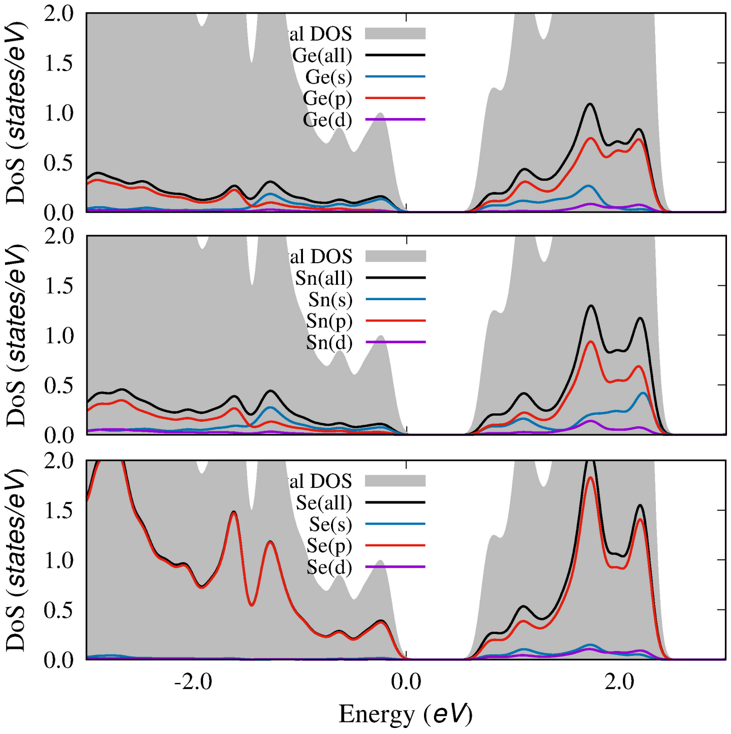


Figure S10: SeGeSnSe internal-Janus: orbital resolved density of states calculated with the PBE exchange-correlation energy functional. The Fermi level is set to zero.

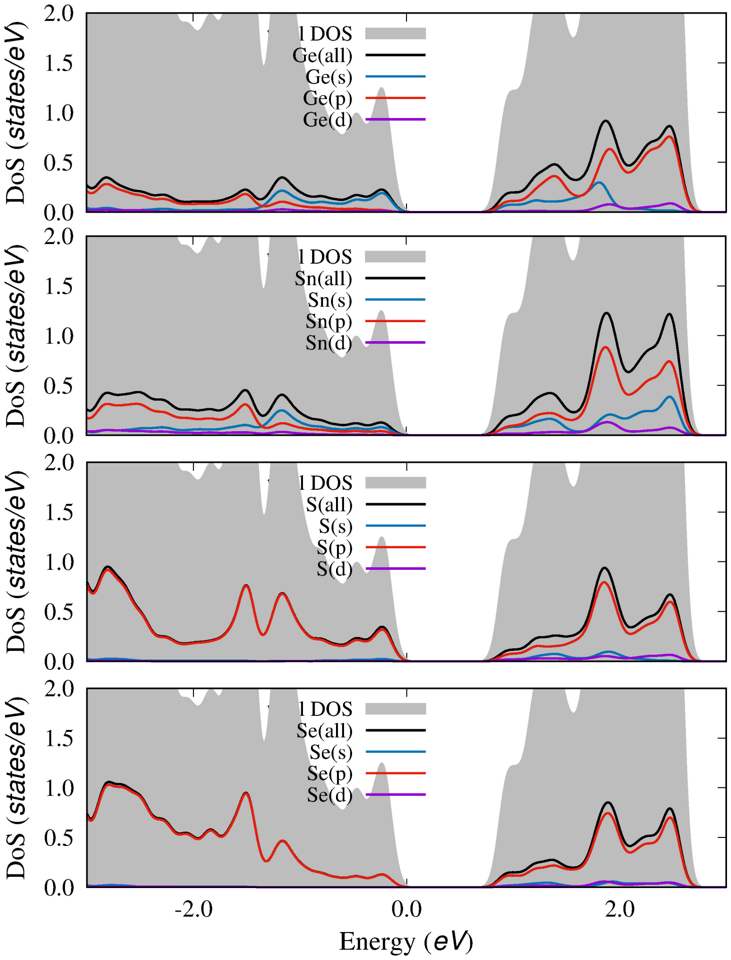


Figure S11: SGeSnSe full-Janus: orbital resolved density of states calculated with the PBE exchange-correlation energy functional. The Fermi level is set to zero.

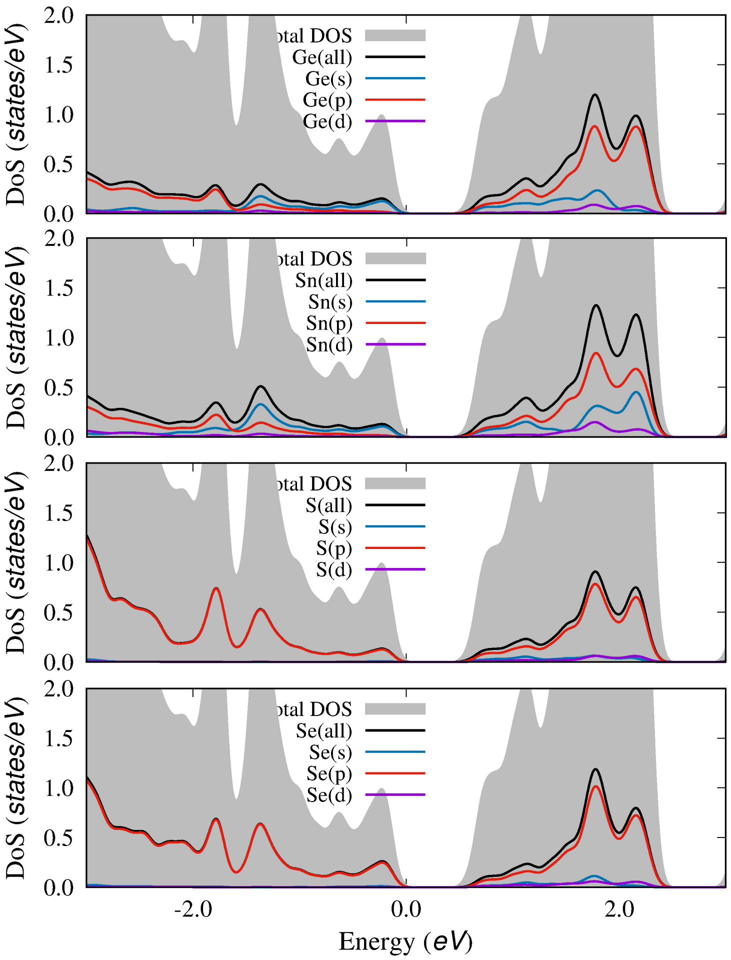


Figure S12: SeGeSnS full-Janus: orbital resolved density of states calculated with the PBE exchange-correlation energy functional. The Fermi level is set to zero.

## S6.2 Spin-Orbit Coupling Effects

Figure S13 contrasts the electronic band structures of P3m1 Janus monolayers for plain PBE and PBE with spin-orbit coupling (PBE+SOC) approaches. Here, the spin-orbit coupling SOC is included using the fully relativistic scheme within the second-order approach in the framework of non-collinear spin density functional theory as implemented in the VASP code.[3] We found a band gap reduction of0.03 eV for SeSnSnSe, which is the system with the heaviest atoms. The highest band gap change is 0.08 eV, occurring for the SeGeSnS Janus monolayer. However, despite these small changes in the band gap, there are splittings around 0.4 eV occurring around 2 eV above the VBM for various compounds.

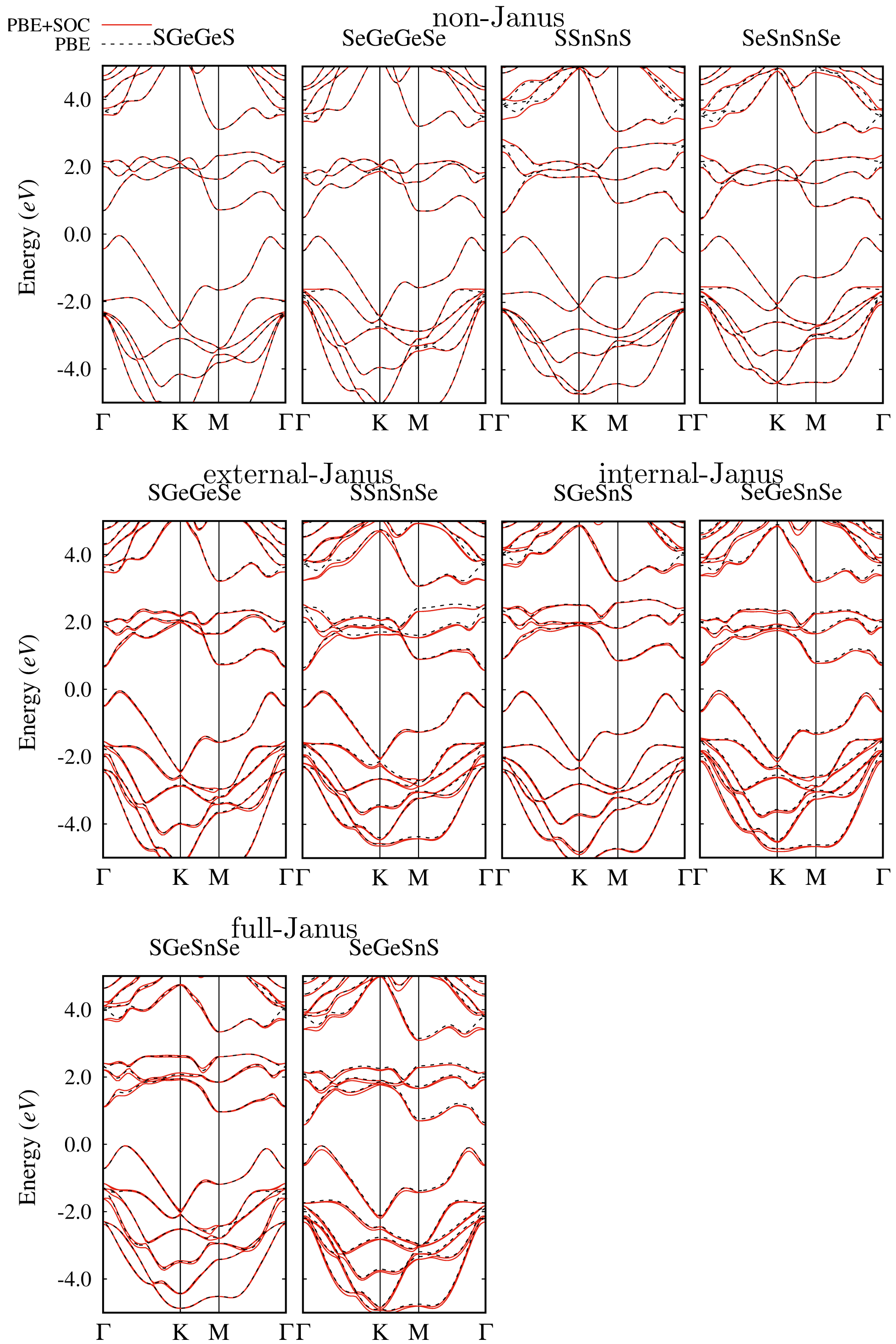


Figure S13: Electronic band structures calculated with spin-orbit-coupling (SOC) in red lines, and without SOC in dashed black lines. These calculations adopts the PBE exchange-correlation energy functional, and the Fermi energy is set to zero in all graphs.

## S6.3 PBE .*vs.* HSE06 electronic band structures

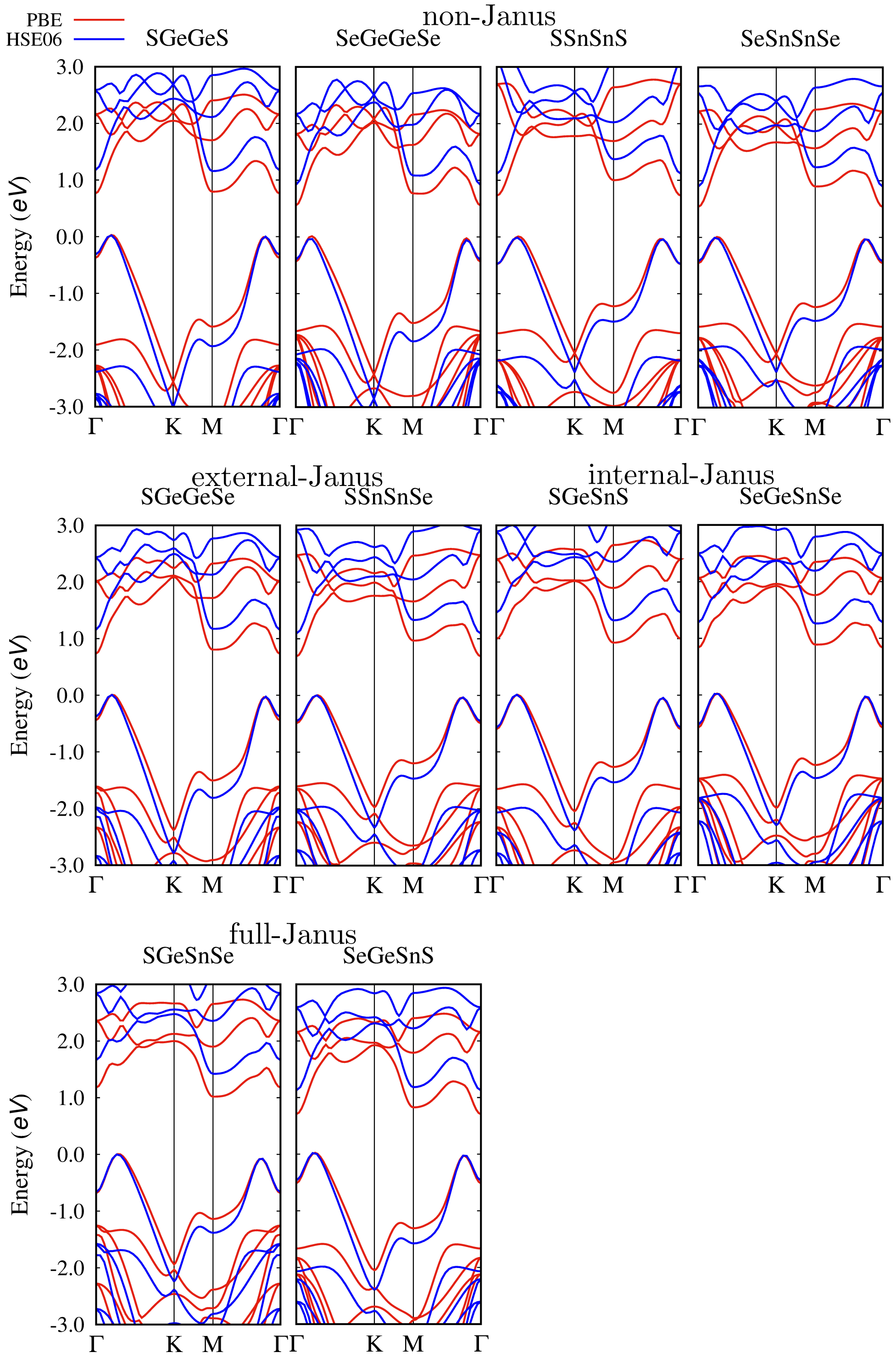


Figure S14: Contrast of the electronic band structures calculated with PBE and HSE06 exchange-correlation energy functionals. The Fermi level is set to zero.

## S6.4 Workfunction

The workfunction is the photon energy necessary for remove one electron from a surface calculated as the energy difference between the vacuum energy and the Fermi level. The vacuum energy is determined from a plateau of the Hartree potential in the vacuum region (between periodic images of monolayers). Due to the non-centrosymmetric character of Janus monolayers, the two monolayer surfaces could present different workfunction vaues due to distinct vacuum energies. Figure S15 depicts the average Hartree potential in a direction perpendicular to the monolayer surfaces. Table S5 present the Fermi energy and the vacuum energies employed in the calculation of the workfunctions.

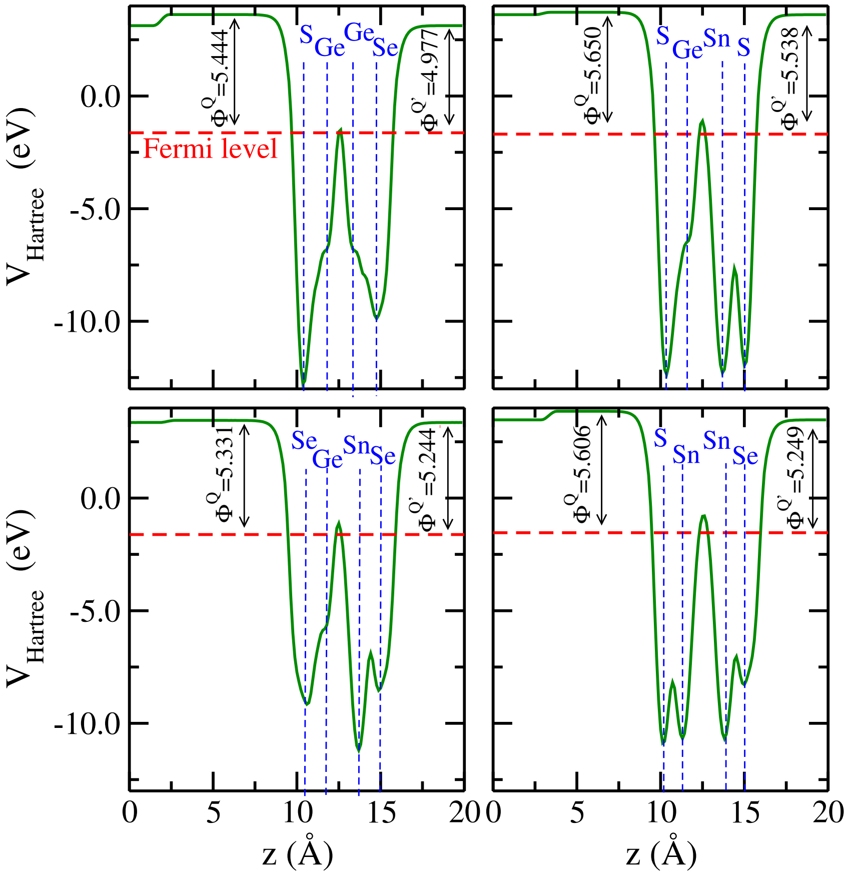


Figure S15: Internal- and external-Janus $QAA'Q'$ compositions: average Hartree potential in the direction perpendicular to monolayer surfaces. The dashed red lines indicate the Fermi level and the blue dashed lines indicate the atomic layers for each specie. In each panel it is also shown workfunctions at the $Q$ ($\Phi^Q$) and $Q'$ ($\Phi^{Q'}$) surfaces in units of eV.

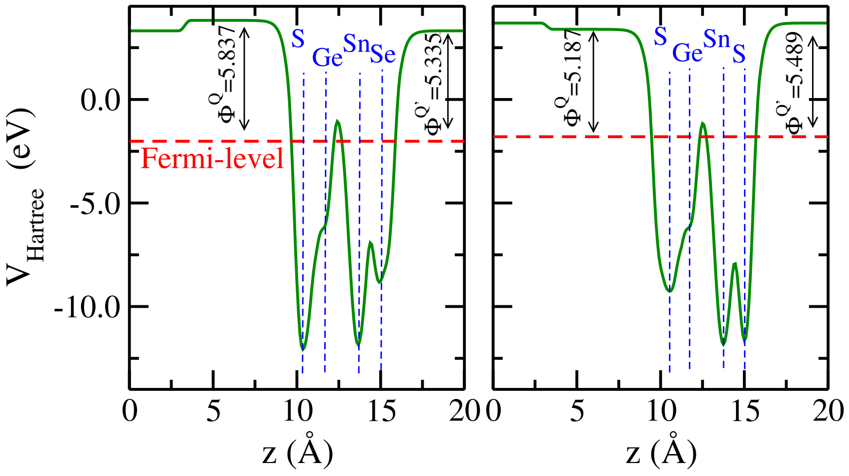


Figure S16: Full-Janus $QAA'Q'$ compositions: average Hartree potential in the direction perpendicular to monolayer surfaces. The dashed red lines indicate the Fermi level and the blue dashed lines indicate the atomic layers for each specie. In each panel it is also shown workfunctions at the $Q$ ($\Phi^Q$) and $Q'$ ($\Phi^{Q'}$) surfaces in units of eV.

Table S5: Energies used for determining the Band offset and workfunctions calculated with the HSE06 exchange-correlation energy functional, namely, energies for the valence band maximum ($E_{\text{VBM}}$) and conduction band minimum ($E_{\text{CBM}}$). Vacuum levels for the $Q$ ($V^Q_{\text{vac.}}$) and $Q'$ ($V^{Q'}_{\text{vac.}}$), their respective workfunctions, $\Phi^Q$ and $\Phi^{Q'}$, and the workfunction variation $\Delta\Phi = \Phi^Q - \Phi^{Q'}$.

| Chem. form. $QAA'Q'$ | $E_{\text{VBM}}$ (eV) | $E_{\text{CBM}}$ (eV) | $V^Q_{\text{vac.}}$ (eV) | $V^{Q'}_{\text{vac.}}$ (eV) | $\Phi^Q$ (eV) | $\Phi^{Q'}$ (eV) | $\Delta\Phi$ (eV) |
|---|---|---|---|---|---|---|---|
| SGeGeS | −1.709 | −0.574 | 3.555 | 3.555 | 5.264 | 5.264 | 0.000 |
| SeGeGeSe | −1.768 | −0.814 | 3.278 | 3.278 | 5.046 | 5.046 | 0.000 |
| SSnSnS | −1.742 | −0.617 | 3.817 | 3.817 | 5.559 | 5.559 | 0.000 |
| SeSnSnSe | −1.668 | −0.767 | 3.553 | 3.553 | 5.221 | 5.221 | 0.000 |
| SGeGeSe | −1.804 | −0.659 | 3.640 | 3.173 | 5.444 | 4.977 | 0.467 |
| SSnSnSe | −1.740 | −0.651 | 3.866 | 3.509 | 5.606 | 5.249 | 0.357 |
| SGeSnS | −1.902 | −0.589 | 3.748 | 3.636 | 5.650 | 5.538 | 0.112 |
| SeGeSnSe | −1.860 | −0.619 | 3.471 | 3.384 | 5.331 | 5.244 | 0.087 |
| SGeSnSe | −2.017 | −0.596 | 3.820 | 3.318 | 5.837 | 5.335 | 0.502 |
| SeGeSnS | −1.799 | −0.693 | 3.388 | 3.690 | 5.187 | 5.489 | −0.302 |

# S7 Wannierization

For the Wannierization procedure, with HSE06 functional, we use the s and p orbital projections for Ge, S and Se, for Sn we use s,p and d projections, the MLWF-TB Hamiltonian basis set is complemented with random projections in order to obtain the same number of bands of

the DFT simulation, this procedure was done with the flag `use_ws_distance=.false.` and `guiding_centres=.true.`.

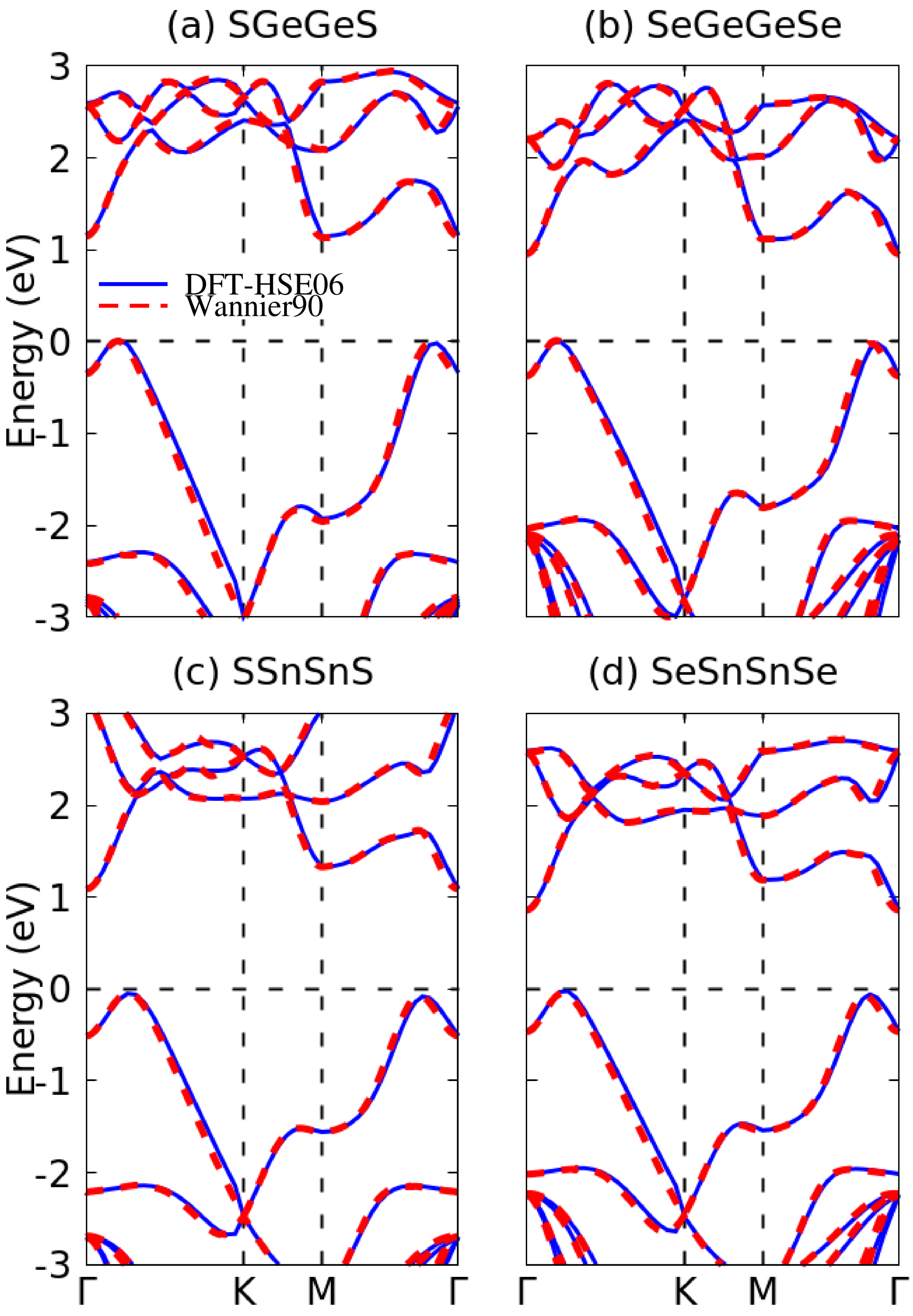


Figure S17: Comparison between DFT-HSE06 (blue solid lines) and MLWF-TB, obtained with Wannier90 (red dashed lines) for non-Janus structures.

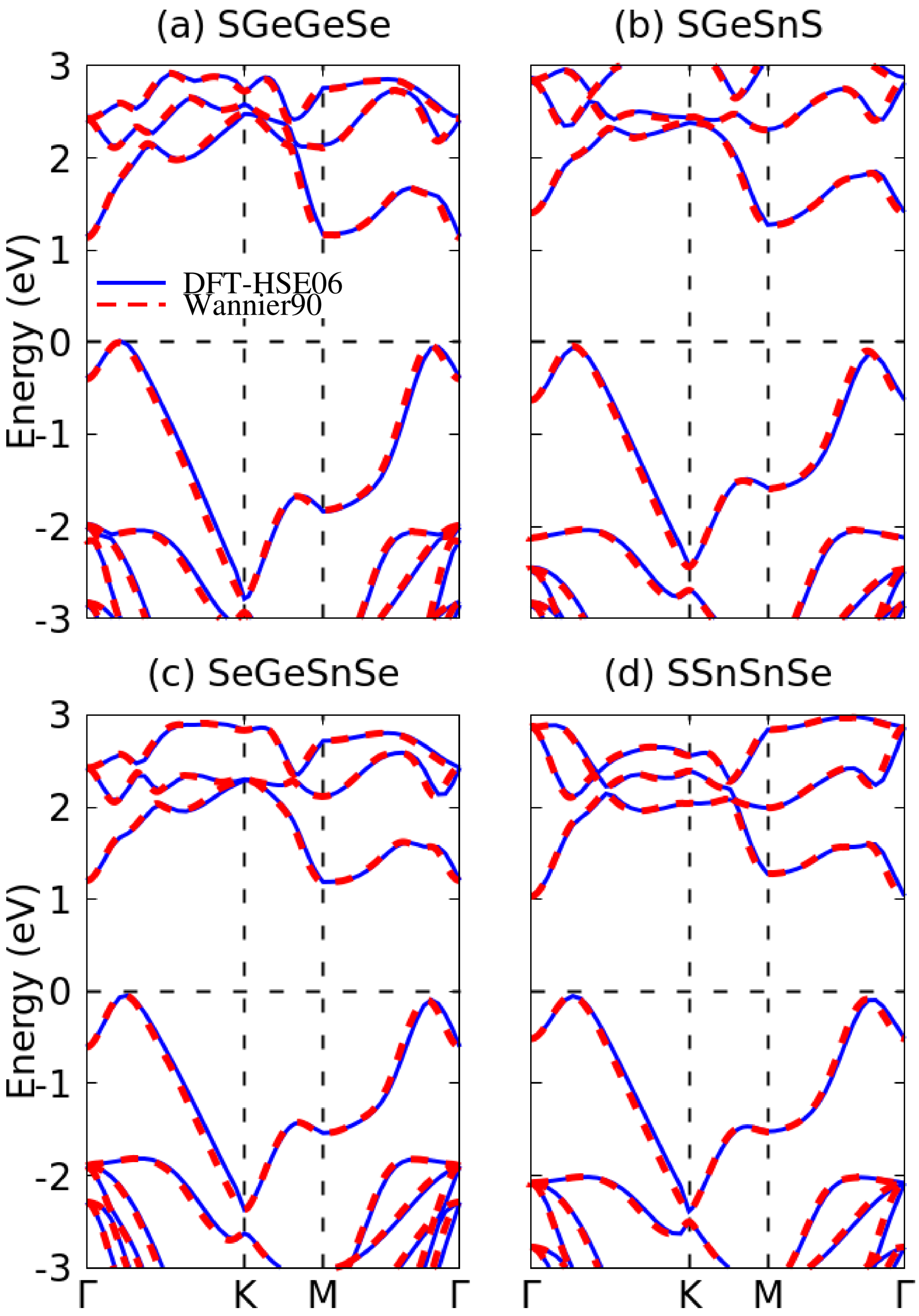


Figure S18: Comparison between DFT-HSE06 (blue solid lines) and MLWF-TB, obtained with Wannier90 (red dashed lines) for external/internal-Janus structures.

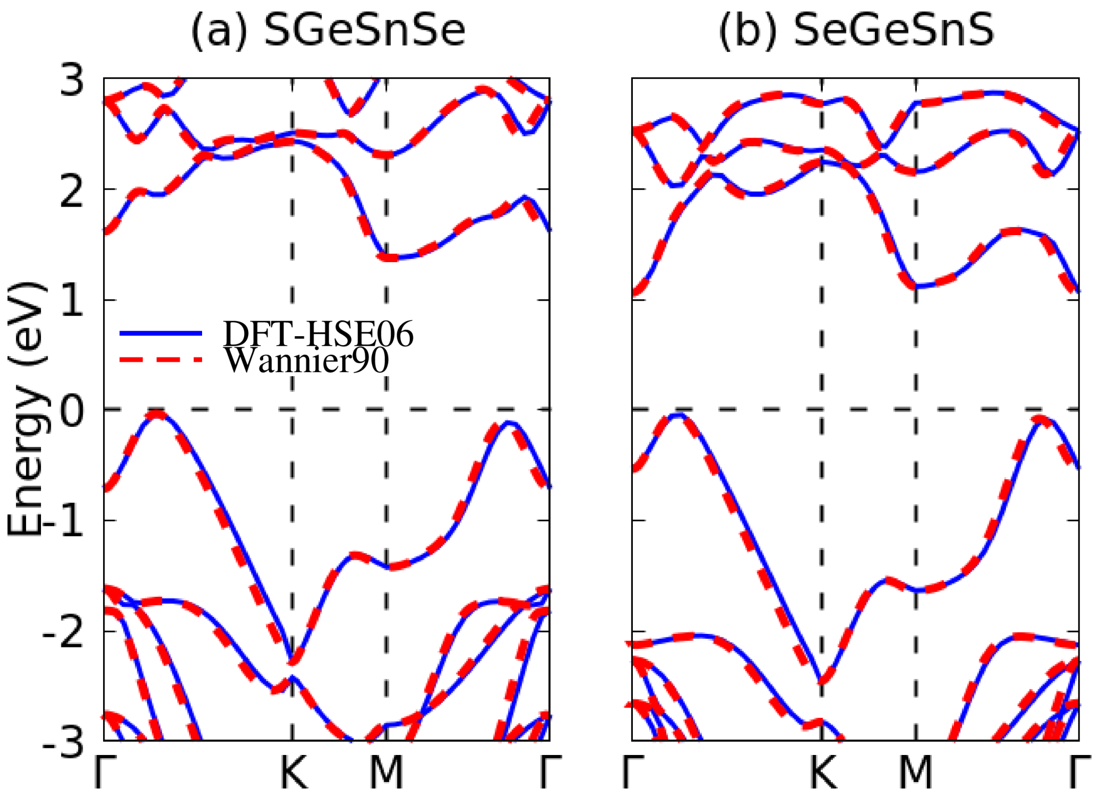


Figure S19: Comparison between DFT-HSE06 (blue solid lines) and MLWF-TB, obtained with Wannier90 (red dashed lines) for full-Janus structures.

## S8 Excitonic and Optical Properties

Table S6: Parameters used for BSE simulations: **k**-points density, $R_k$ and their correspondent **k**-mesh, $n_v$, number of valence bands, $n_c$, number of conduction bands, in order to get all optical transitions in the solar emission spectrum range. i.e 0.5 eV to 4.0 eV and dielectric function smearing $\eta$. All simulations were done using a Coulomb truncated 2D potential (V2DT),[4] implemented in WanTiBEXOS package.[5]

| Chem. form. | $R_k$ | **k**-mesh | $n_v$ | $n_c$ | $\eta$ (eV) |
|---|---|---|---|---|---|
| SGeGeS | 120 | $38 \times 38 \times 1$ | 6 | 3 | 0.05 |
| SeGeGeSe | 120 | $36 \times 36 \times 1$ | 6 | 3 | 0.05 |
| SSnSnS | 120 | $35 \times 35 \times 1$ | 6 | 3 | 0.05 |
| SeSnSnSe | 120 | $34 \times 34 \times 1$ | 6 | 3 | 0.05 |
| SGeGeSe | 120 | $37 \times 37 \times 1$ | 6 | 3 | 0.05 |
| SSnSnSe | 120 | $34 \times 34 \times 1$ | 6 | 3 | 0.05 |
| SGeSnS | 120 | $36 \times 36 \times 1$ | 3 | 3 | 0.05 |
| SeGeSnSe | 120 | $35 \times 35 \times 1$ | 3 | 4 | 0.05 |
| SGeSnSe | 120 | $36 \times 36 \times 1$ | 3 | 3 | 0.05 |
| SeGeSnS | 120 | $36 \times 36 \times 1$ | 5 | 3 | 0.05 |

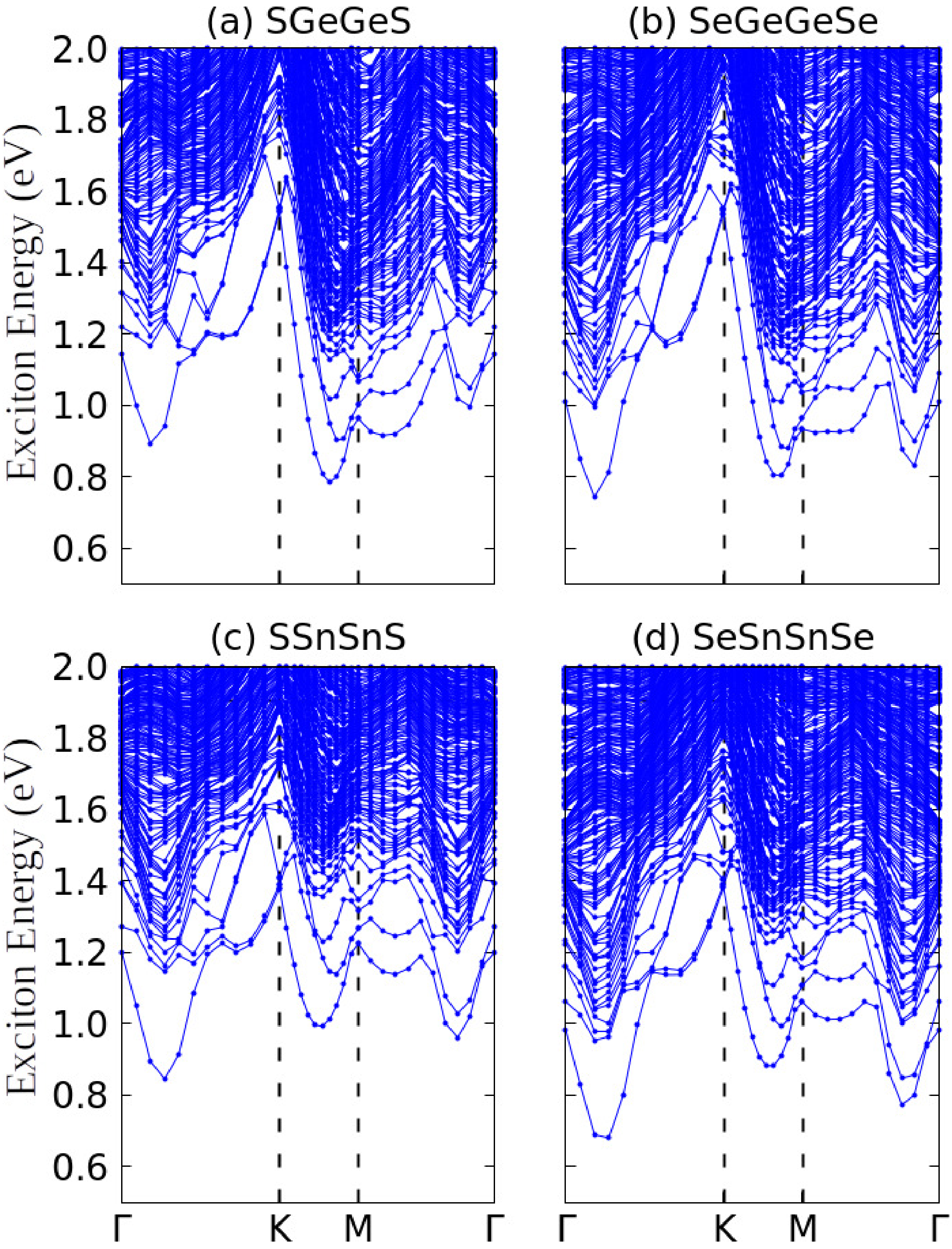


Figure S20: Exciton band structure obtained with MLWF-TB+BSE for non-Janus structures.

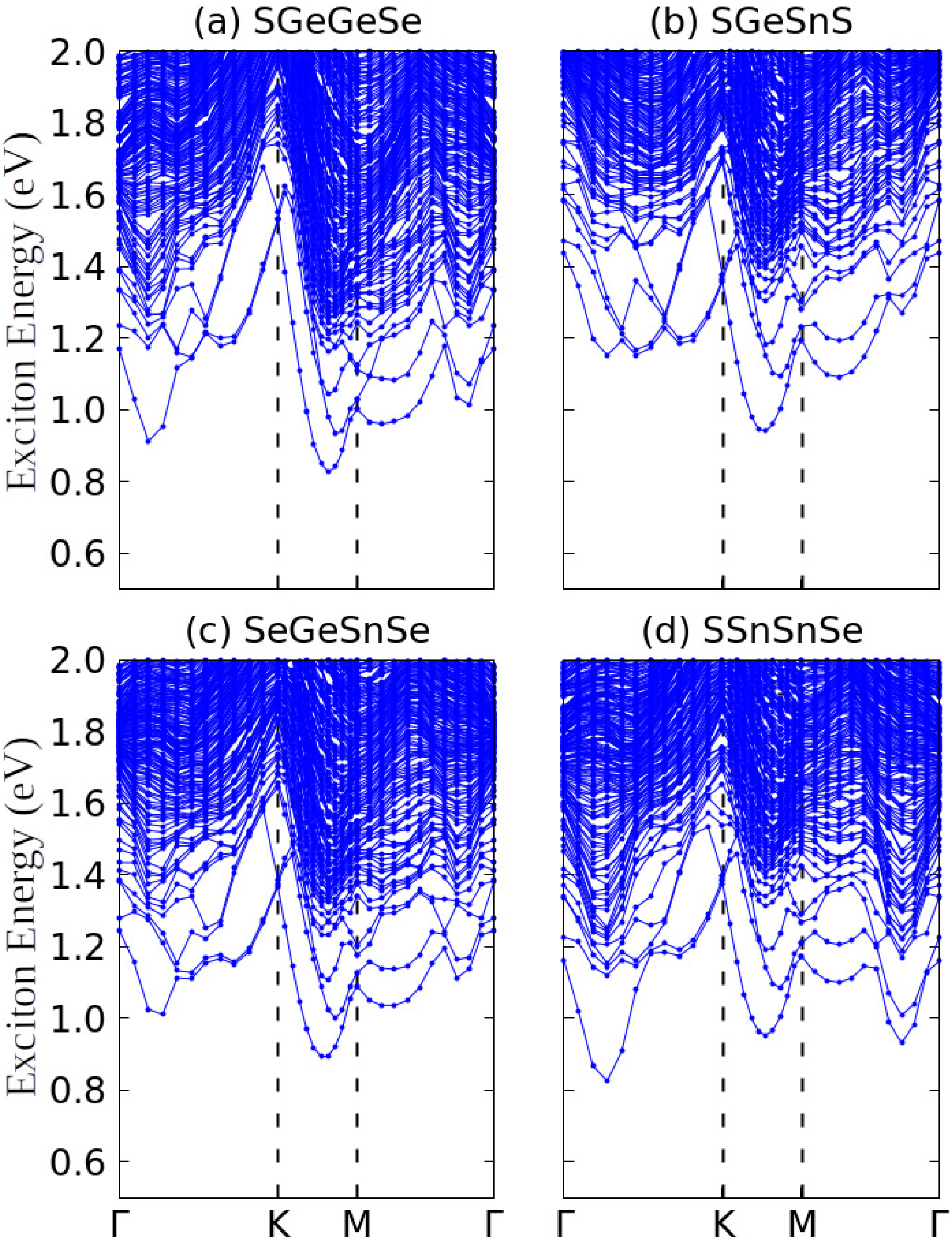


Figure S21: Exciton band structure obtained with MLWF-TB+BSE for external/internal-Janus structures.

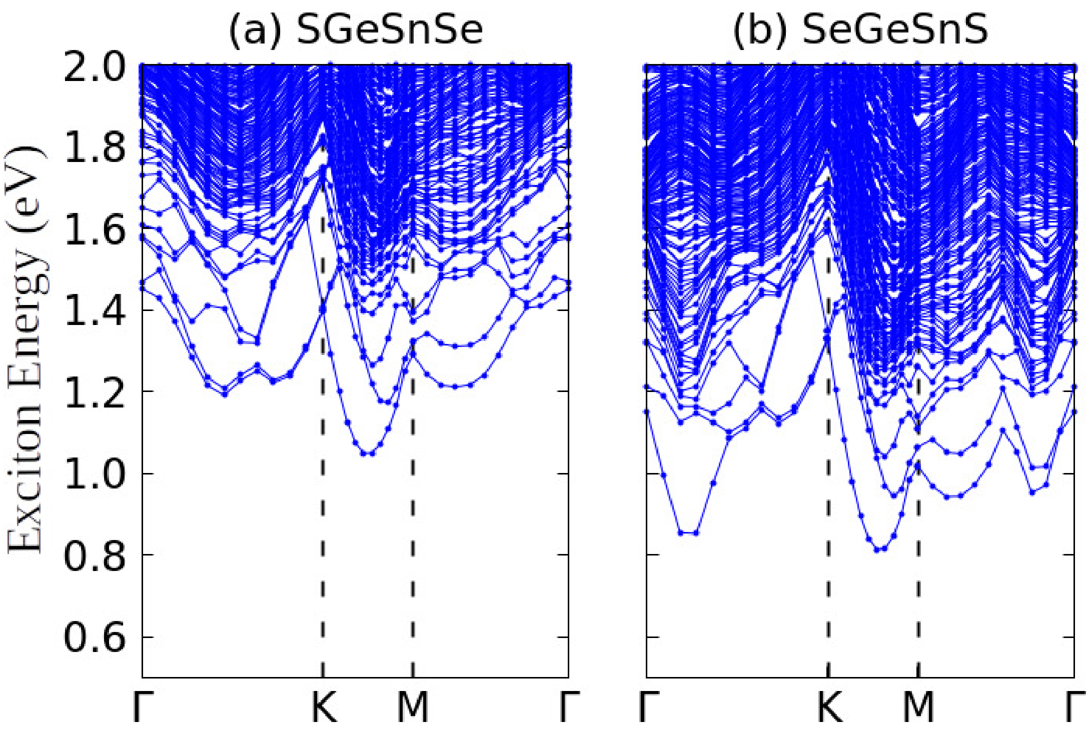


Figure S22: Exciton band structure obtained with MLWF-TB+BSE for full-Janus structures.

Table S7: MLWF-TB+BSE calculated excitonic properties: fundamental band gap, $E_g$ , direct band gap, $E_g^d$ , exciton ground state, $Ex_{gs}$ , direct exciton ground state, $Ex_{gs}^d$ , and exciton binding energy, $Ex_b$, obtained from $E_g - Ex_{gs}$. All direct excitons ground states are bright.

| Chem. form. | $E_g$ (eV) | $E_g^d$ (eV) | $Ex_{gs}$ (eV) | $Ex_{gs}^d$ (eV) | $Ex_b$ (eV) |
|---|---|---|---|---|---|
| SGeGeS | 1.13 | 1.49 | 0.77 | 1.14 | 0.36 |
| SeGeGeSe | 0.94 | 1.32 | 0.74 | 1.01 | 0.20 |
| SSnSnS | 1.13 | 1.60 | 0.84 | 1.20 | 0.29 |
| SeSnSnSe | 0.90 | 1.32 | 0.68 | 0.98 | 0.22 |
| SGeGeSe | 1.14 | 1.54 | 0.83 | 1.17 | 0.31 |
| SSnSnSe | 1.08 | 1.55 | 0.83 | 1.16 | 0.25 |
| SGeSnS | 1.31 | 1.92 | 0.94 | 1.44 | 0.37 |
| SeGeSnSe | 1.23 | 1.66 | 0.89 | 1.24 | 0.34 |
| SGeSnSe | 1.41 | 1.92 | 1.05 | 1.45 | 0.36 |
| SeGeSnS | 1.10 | 1.57 | 0.81 | 1.15 | 0.29 |

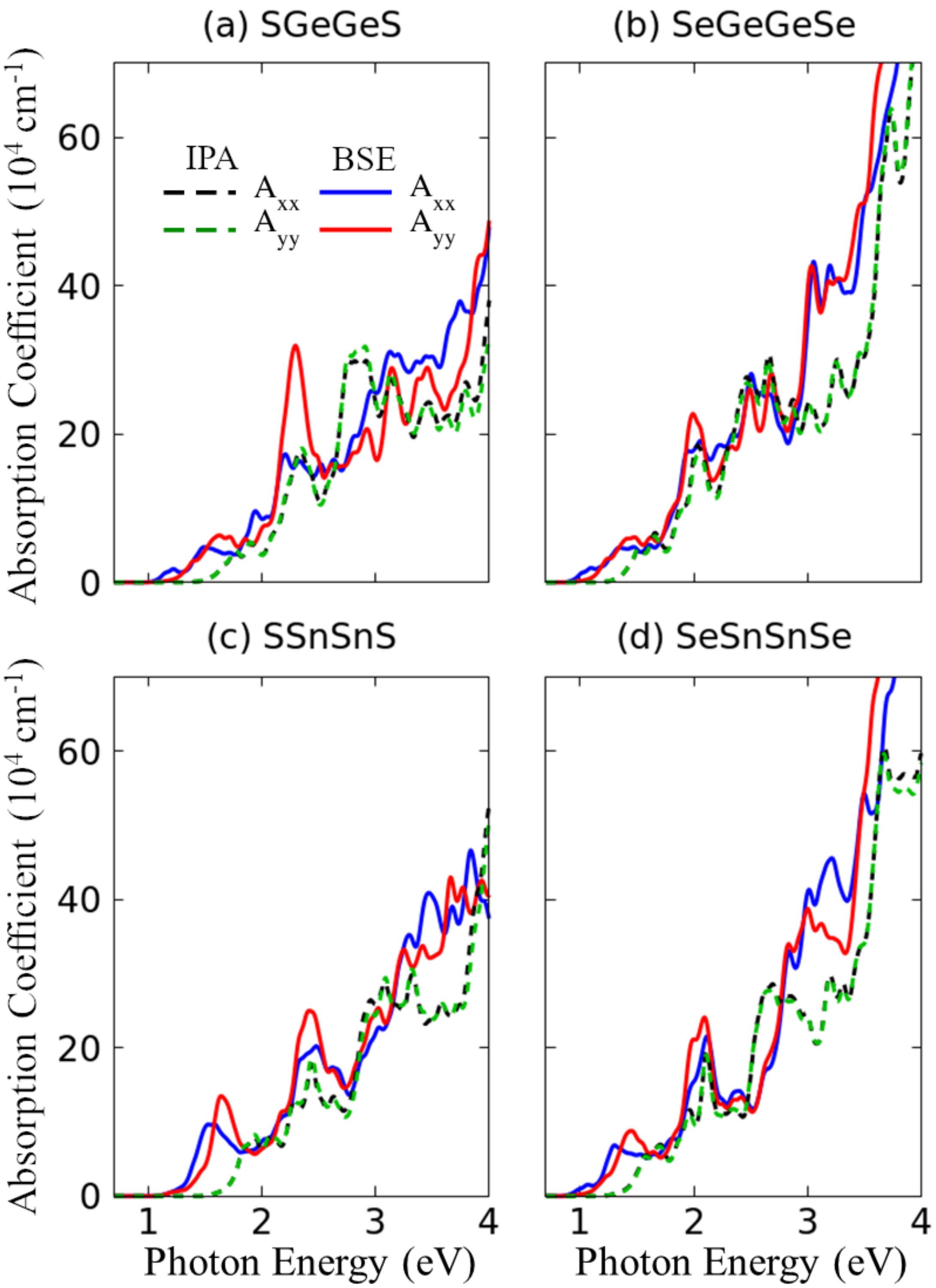


Figure S23: Absorption Coefficient, considering linear light polarization at $\hat{x}$ and $\hat{y}$ at BSE (solid lines) and IPA (dashed lines) levels for non-Janus structures.

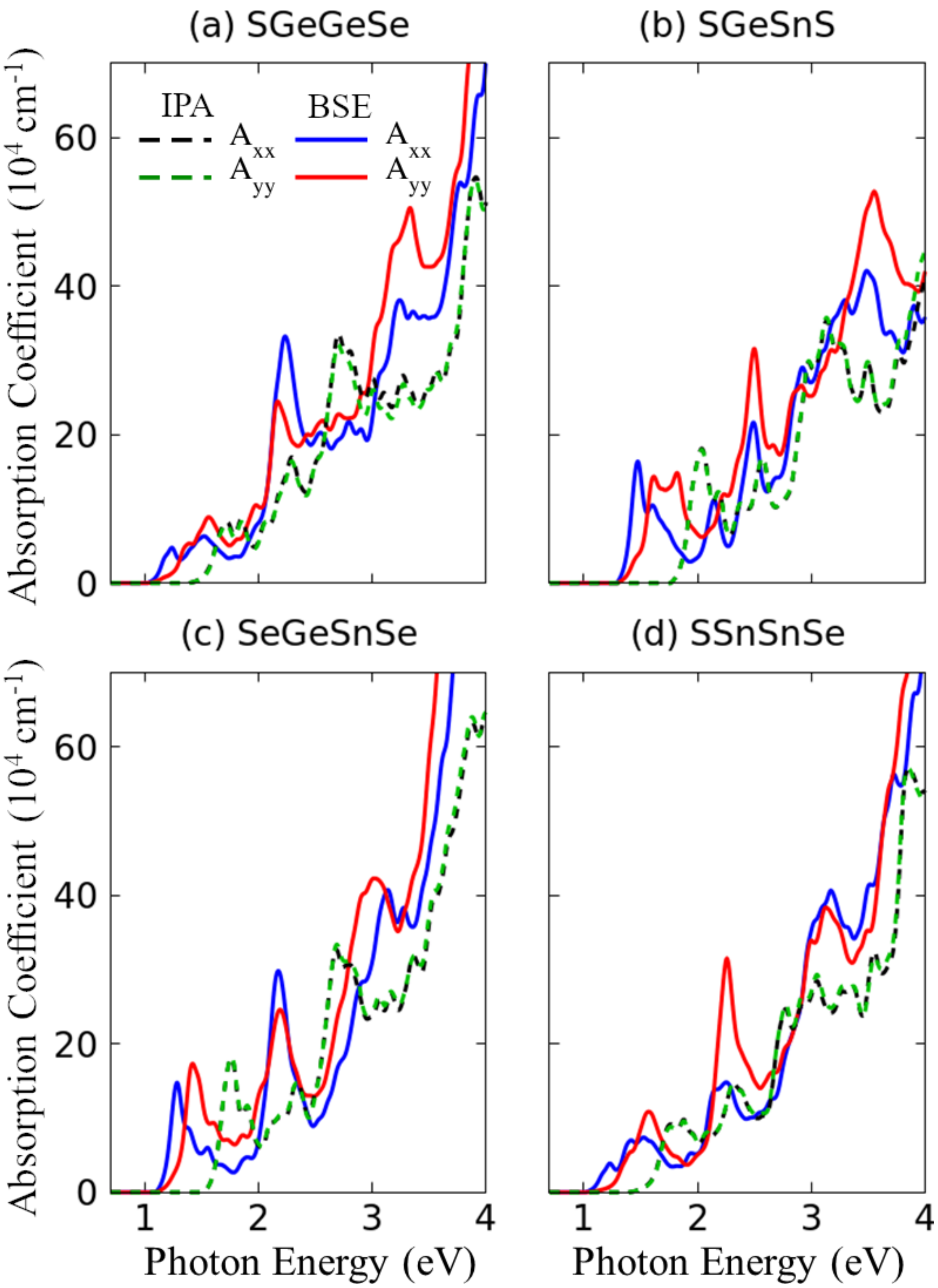


Figure S24: Absorption Coefficient, considering linear light polarization at $\hat{x}$ and $\hat{y}$ at BSE (solid lines) and IPA (dashed lines) levels for external/internal-Janus structures.

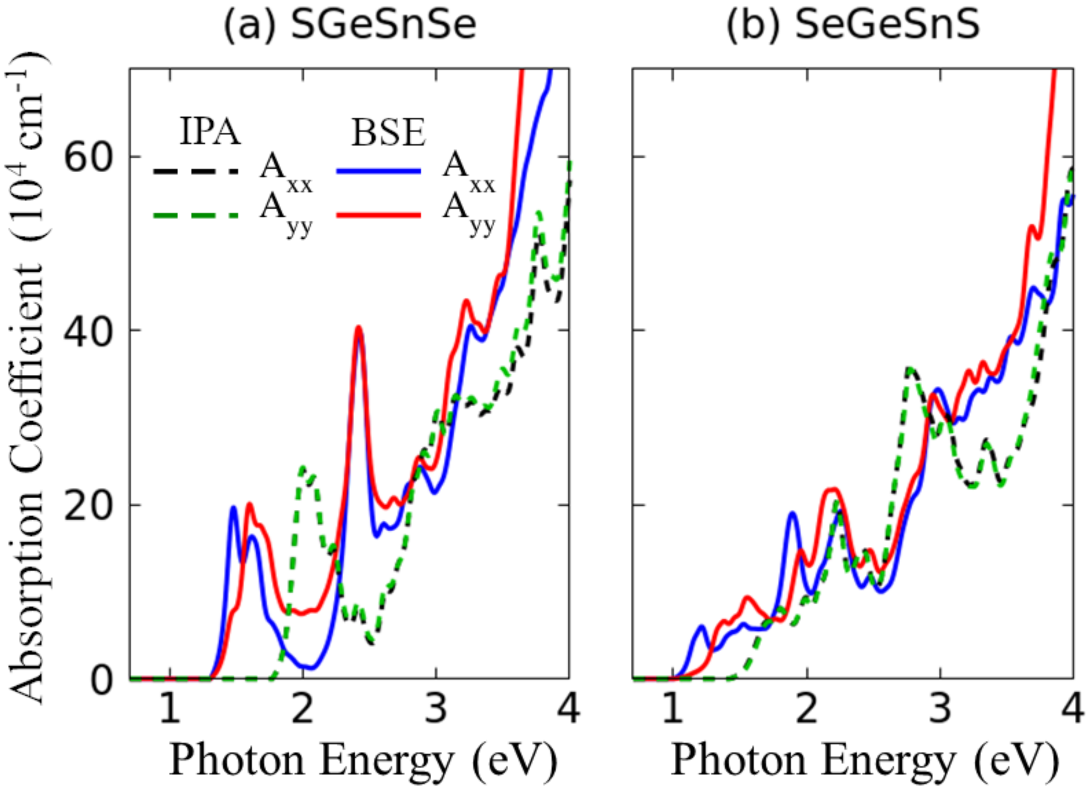


Figure S25: Absorption Coefficient, considering linear light polarization at $\hat{x}$ and $\hat{y}$ at BSE (solid lines) and IPA (dashed lines) levels for full-Janus structures.

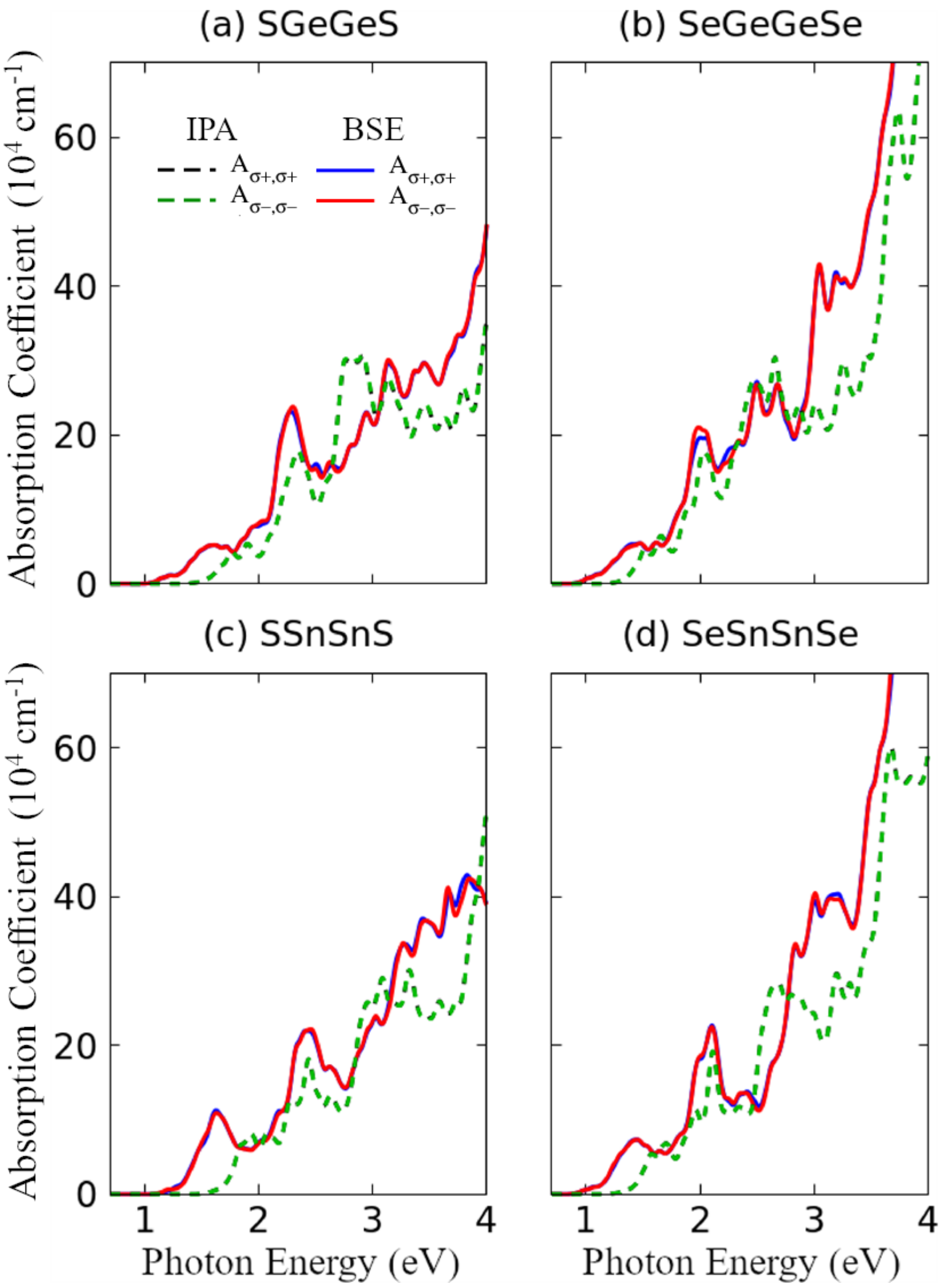


Figure S26: Absorption Coefficient, considering circular light polarization, $\sigma^+$ and $\sigma^-$ at BSE (solid lines) and IPA (dashed lines) levels for non-Janus structures.

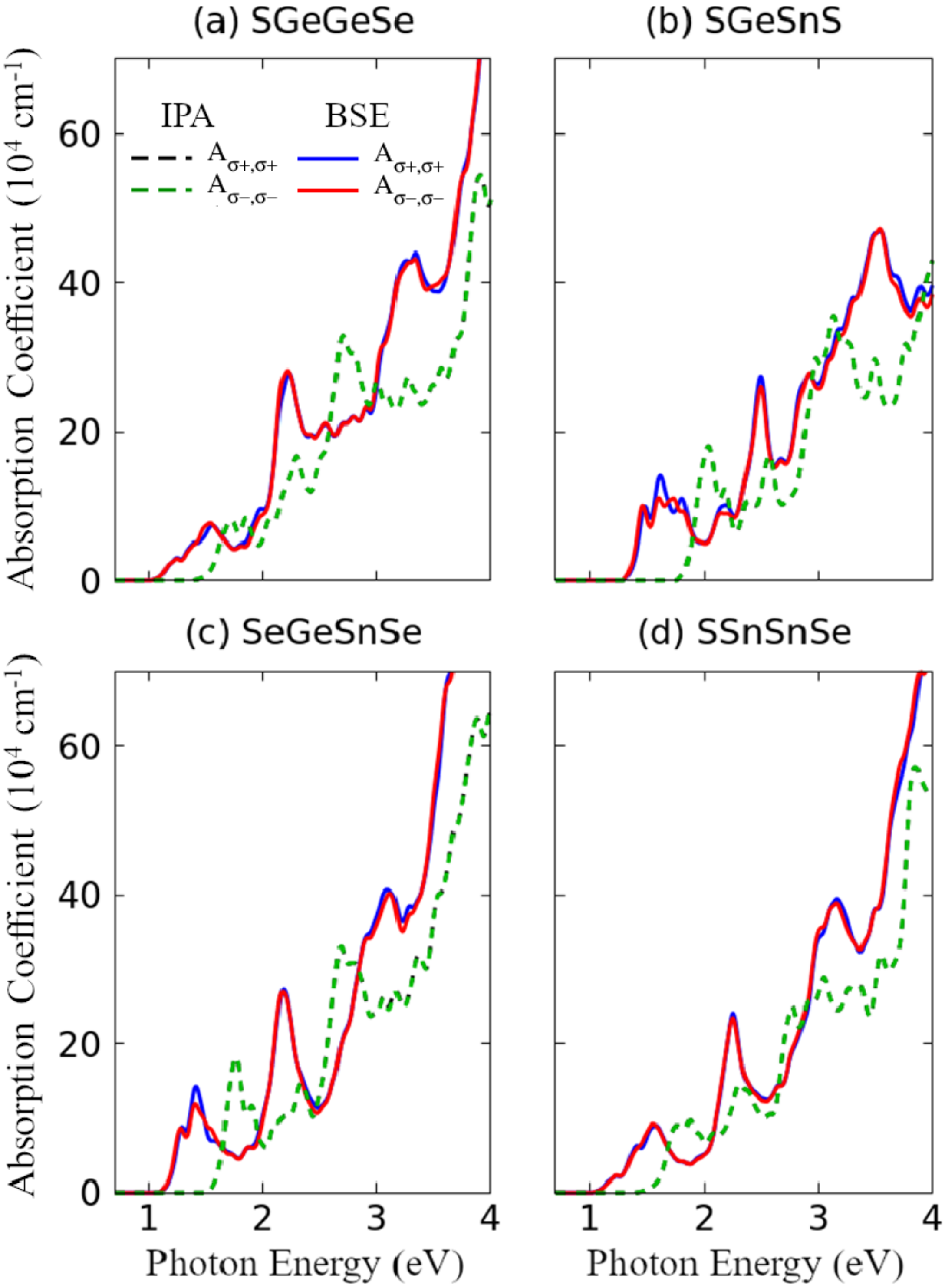


Figure S27: Absorption Coefficient, considering circular light polarization, $\sigma^+$ and $\sigma^-$ at BSE (solid lines) and IPA (dashed lines) levels for external/internal-Janus structures.

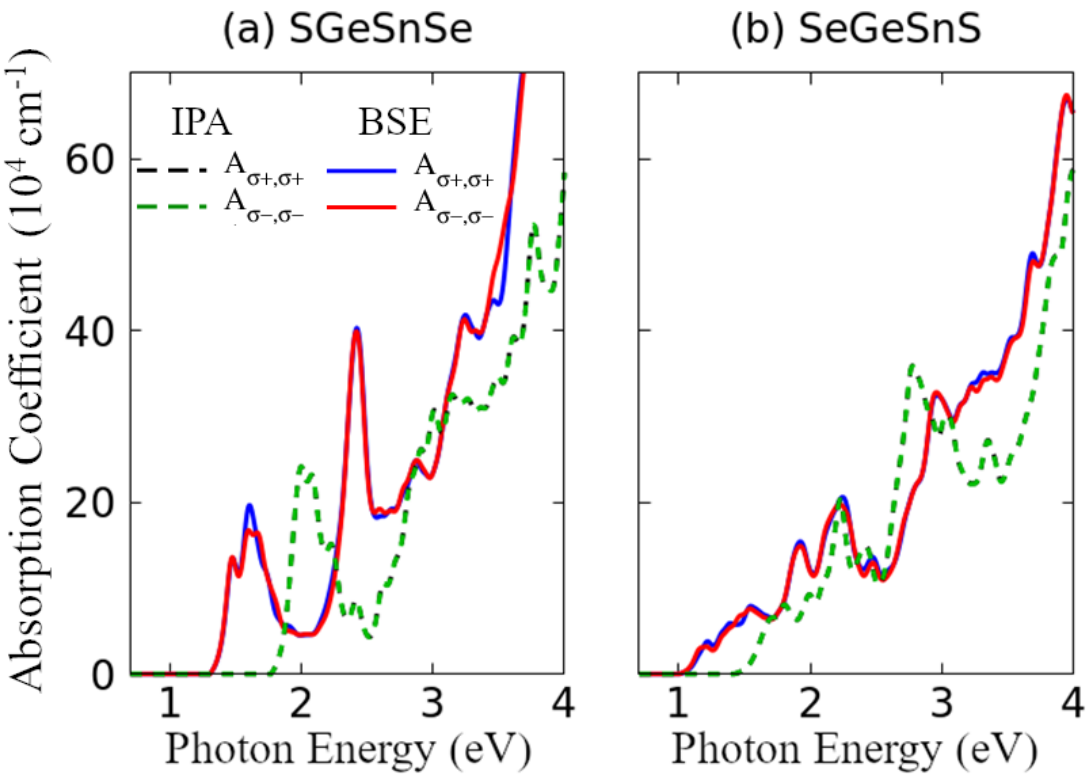


Figure S28: Absorption Coefficient, considering circular light polarization, $\sigma^+$ and $\sigma^-$ at BSE (solid lines) and IPA (dashed lines) levels for full-Janus structures.

Table S8: Maximum achieved PCE at IPA level, short circuit current density, $J_{sc}$ (W/Vm$^2$), open circuit voltage, $V_{oc}$ (V), $fr$ recombination fraction, $FF$ fill factor (%), monolayer thickness plus vdW length (3.21 Å), Δ (Å), power conversion efficiency obtained by SLME, PCE$^{\text{SLME}}$ (%), power conversion efficiency obtained by SLME considering that 100 % of photon absorbance starts from direct band gap, PCE$^{\text{SLME}}_{max}$ (%), and obtained in Shockley–Queisser limit (considering direct band gap), PCE$^{\text{SQ}}$ (%), calculated with $T = 298.15$ K.

| System | $J_{sc}$ | $V_{oc}$ | $-lnfr$ | $FF$ | Δ | PCE$^{\text{SLME}}$ | PCE$^{\text{SLME}}_{max}$ | PCE$^{\text{SQ}}$ |
|---|---|---|---|---|---|---|---|---|
| SGeGeS | 8.44 | 0.91 | 14.25 | 87.40 | 8.09 | 0.67 | 20.99 | 31.35 |
| SeGeGeSe | 13.37 | 0.72 | 15.00 | 85.02 | 8.24 | 0.82 | 19.44 | 32.61 |
| SSnSnS | 8.26 | 0.90 | 18.10 | 87.33 | 8.64 | 0.65 | 18.33 | 28.88 |
| SeSnSnSe | 12.69 | 0.70 | 16.36 | 84.60 | 8.76 | 0.75 | 18.19 | 32.55 |
| SGeGeSe | 10.55 | 0.89 | 15.37 | 87.17 | 8.15 | 0.82 | 20.26 | 30.93 |
| SSnSnSe | 10.00 | 0.84 | 18.04 | 86.62 | 8.69 | 0.73 | 18.43 | 30.77 |
| SGeSnS | 7.85 | 1.00 | 23.59 | 88.25 | 8.34 | 0.69 | 14.40 | 24.21 |
| SeGeSnSe | 12.13 | 0.93 | 16.92 | 87.60 | 8.50 | 0.99 | 19.04 | 29.06 |
| SGeSnSe | 9.10 | 1.09 | 19.79 | 89.01 | 8.42 | 0.88 | 15.97 | 24.21 |
| SeGeSnS | 11.03 | 0.84 | 18.26 | 86.59 | 8.41 | 0.80 | 18.27 | 30.42 |

Table S9: Maximum achieved PCE at BSE level, short circuit current density, $J_{sc}$ (W/Vm$^2$), open circuit voltage, $V_{oc}$ (V), $fr$ recombination fraction, $FF$ fill factor (%), monolayer thickness plus vdW length (3.21 Å), $\Delta$ (m), power conversion efficiency obtained by SLME, PCE$^{\text{SLME}}$ (%), power conversion efficiency obtained by SLME considering that 100 % of photon absorbance starts from exciton bright ground state, PCE$^{\text{SLME}}_{max}$ (%), and obtained in Shockley–Queisser limit (considering exciton bright ground state), PCE$^{\text{SQ}}$ (%), calculated with $T = 298.15$ K.

| System | $J_{sc}$ | $V_{oc}$ | $-lnfr$ | $FF$ | $\Delta$ | PCE$^{\text{SLME}}$ | PCE$^{\text{SLME}}_{max}$ | PCE$^{\text{SQ}}$ |
|---|---|---|---|---|---|---|---|---|
| SGeGeS | 11.51 | 0.57 | 13.93 | 82.27 | 8.09 | 0.54 | 17.72 | 32.36 |
| SeGeGeSe | 16.91 | 0.54 | 10.38 | 81.58 | 8.24 | 0.75 | 18.22 | 30.34 |
| SSnSnS | 13.13 | 0.64 | 13.85 | 83.60 | 8.76 | 0.70 | 18.63 | 32.29 |
| SeSnSnSe | 17.03 | 0.49 | 11.75 | 80.12 | 8.76 | 0.67 | 16.04 | 30.14 |
| SGeGeSe | 14.76 | 0.59 | 13.24 | 82.71 | 8.15 | 0.72 | 18.81 | 32.37 |
| SSnSnSe | 13.62 | 0.60 | 12.99 | 82.76 | 8.69 | 0.67 | 18.93 | 32.37 |
| SGeSnS | 12.78 | 0.66 | 19.40 | 83.95 | 8.34 | 0.71 | 16.98 | 32.06 |
| SeGeSnSe | 16.59 | 0.63 | 13.70 | 83.35 | 8.50 | 0.86 | 19.24 | 32.07 |
| SGeSnSe | 15.30 | 0.76 | 15.68 | 85.60 | 8.42 | 1.00 | 19.88 | 31.89 |
| SeGeSnS | 15.20 | 0.57 | 13.10 | 82.28 | 8.41 | 0.72 | 18.67 | 32.37 |

# References


1 Xiong, F.; Zhang, X.; Lin, Z.; Chen, Y. Ferroelectric engineering of two-dimensional group-IV monochalcogenides: The effects of alloying and strain. *J. Materiomics* **2018**, *4*, 139–143, DOI: 10.1016/j.jmat.2018.02.005, Two Dimensional Materials.

2 Sa, B.; Sun, Z.; Wu, B. The development of two dimensional group IV chalcogenides, blocks for van der Waals heterostructures. *Nanoscale* **2016**, *8*, 1169–1178, DOI: 10.1039/C5NR06871A.

3 Steiner, S.; Khmelevskyi, S.; Marsmann, M.; Kresse, G. Calculation of the magnetic anisotropy with projected-augmented-wave methodology and the case study of disordered $Fe_{1-x}Co_x$ alloys. *Phys. Rev. B* **2016**, *93*, 224425, DOI: 10.1103/PhysRevB.93.224425.

4 Rozzi, C. A.; Varsano, D.; Marini, A.; Gross, E. K. U.; Rubio, A. Exact Coulomb cutoff technique for supercell calculations. *Phys. Rev. B* **2006**, *73*, 205119, DOI: 10.1103/PhysRevB.73.205119.

5 Dias, A. C.; Silveira, J. F.; Qu, F. WanTiBEXOS: A Wannier based Tight Binding code for

electronic band structure, excitonic and optoelectronic properties of solids. *Comput. Phys. Commun.* **2023**, *285*, 108636, DOI: 10.1016/j.cpc.2022.108636.
